# Reprogramming of Murine Macrophages through TLR2 Confers Viral Resistance via TRAF3-Mediated, Enhanced Interferon Production

**DOI:** 10.1371/journal.ppat.1003479

**Published:** 2013-07-11

**Authors:** Darren J. Perkins, Swamy K. Polumuri, Meghan E. Pennini, Wendy Lai, Ping Xie, Stefanie N. Vogel

**Affiliations:** 1 Department of Microbiology and Immunology, University of Maryland, Baltimore (UMB), School of Medicine, Baltimore, Maryland, United States of America; 2 Department of Cell Biology and Neuroscience, Rutgers University, Piscataway, New Jersey, United States of America; University of North Carolina at Chapel Hill, United States of America

## Abstract

The cell surface/endosomal Toll-like Receptors (TLRs) are instrumental in initiating immune responses to both bacteria and viruses. With the exception of TLR2, all TLRs and cytosolic RIG-I-like receptors (RLRs) with known virus-derived ligands induce type I interferons (IFNs) in macrophages or dendritic cells. Herein, we report that prior ligation of TLR2, an event previously shown to induce “homo” or “hetero” tolerance, strongly “primes” macrophages for increased Type I IFN production in response to subsequent TLR/RLR signaling. This occurs by increasing activation of the transcription factor, IFN Regulatory Factor-3 (IRF-3) that, in turn, leads to enhanced induction of IFN-β, while expression of other pro-inflammatory genes are suppressed (tolerized). *In vitro* or *in vivo* “priming” of murine macrophages with TLR2 ligands increase virus-mediated IFN induction and resistance to infection. This priming effect of TLR2 is mediated by the selective upregulation of the K63 ubiquitin ligase, TRAF3. Thus, we provide a mechanistic explanation for the observed antiviral actions of MyD88-dependent TLR2 and further define the role of TRAF3 in viral innate immunity.

## Author Summary

In response to viral infection, cells of the innate immune system synthesize and release members of the type I interferon protein family. The interferons form an essential line of defense, both by slowing viral growth and by expanding the cellular immune response. The synthesis of interferon is initiated by recognition of viral constituents by one or more innate receptors. Among these receptors, Toll like receptor 2 (TLR2) has been shown to be critical for the immune response to a number of viruses, yet TLR2 only directly initiates Type I interferon production in a very small set of innate immune cells. We have discovered that TLR 2 can contribute to the antiviral interferon response much more broadly by indirectly governing the production of interferon induced by other Toll like receptors as wells as downstream of the cytosolic Rig-I like receptors. This happens through the TLR2-dependent up-regulation of a critical signaling element, TRAF3. We also demonstrate that this TLR2 dependent regulation of interferon may be important in biological scenarios involving co-infection of virus and Gram positive bacteria, but not Gram negative bacteria.

## Introduction

The last few years have seen an explosion in the characterization of mechanisms for the recognition of microbial pathogens by the innate immune system. In particular, sensors that recognize molecular signatures of viral infection have been the subject of many exciting discoveries. Among the currently known innate immune antiviral sensors are the cytosolic RNA receptors, Retinoic acid-inducible gene 1 (RIG-I), and Melanoma differentiation-associated protein 5 (MDA5) [1]–[3], as well as, DDX21 and DHX36 (DDX/TRIF) [4]. A cytosolic DNA sensing multi-protein complex has been identified that responds to DNA virus infections, although the apical sensors for this pathway have not been fully elucidated [5], [6]. In addition, the nucleic acid sensing endosomal Toll-like receptors (TLRs), *i.e.*, TLR3, TLR7/8, and TLR9, as well as cell surface expressed TLR4, have known virus-derived ligands [7], [8]. A common and critical feature of each of these innate viral surveillance systems is the ability to induce type I interferons (IFNs). IFNs are a family of pleotropic cytokines that are secreted and act in a paracrine or autocrine manner to induce an incredibly diverse array of genes with direct antiviral properties [9], [10]. Additionally, type I IFNs serve as a point of connection between innate and adaptive responses, in that they educate and enhance the adaptive response and lead to viral clearance [11].

Among the TLRs, TLRs 2 and 5 are unique in that they utilize MyD88-dependent signaling exclusively and, thus, do not induce type I IFNs in macrophages and dendritic cells [12]. As a result, these receptors are generally described as being antibacterial. While there are no known virus-derived ligands for TLR5, there are a number of reports in the literature describing TLR2 activation by viral ligands, including a recent report that has shown that viral, but not bacterial, TLR2 ligands may induce IFN in a subset of monocytes by unknown mechanisms [13]. In addition, mouse infection models with several different viruses have shown deficiencies in viral clearance in mice with a targeted deletion in TLR2 [14]–[16]. Presently, the mechanism by which TLR2 contributes to an antiviral state in the absence of direct IFN induction is not clear.

Along with the well documented role that individual TLR and RLR sensors play in responding to infection, it is becoming increasingly clear that individual innate immune sensing systems do not operate in isolation, but that significant cross-talk between innate systems occurs [17]. Indeed, the biological relevance of such cross-talk has already been demonstrated in cases of polymicrobial infection [18], [19].

Herein, we describe a TLR2-dependent mechanism for the governance of subsequent type I IFN production via both the TLR and RLR systems. This pathway shapes antiviral immunity *in vitro* in murine primary macrophages, and *in vivo* in mouse models of viral infection. In response to prior stimulation or “priming” with TLR2 ligands, subsequent type I IFN induction via all known IFN-β-inducing innate immune pathways is strongly potentiated. The underlying mechanism for this potentiation was identified as being largely due to the up-regulation of the E3 ubiquitin ligase, TRAF3. These findings not only explain how bacterial or viral TLR2 ligands may selectively augment a subsequent TLR-mediated IFN response to virus, but also reveal a new degree of mechanistic cooperativity between TLRs and the cytosolic RLRs in the host response to virus infection.

## Results

**Figure 1:**
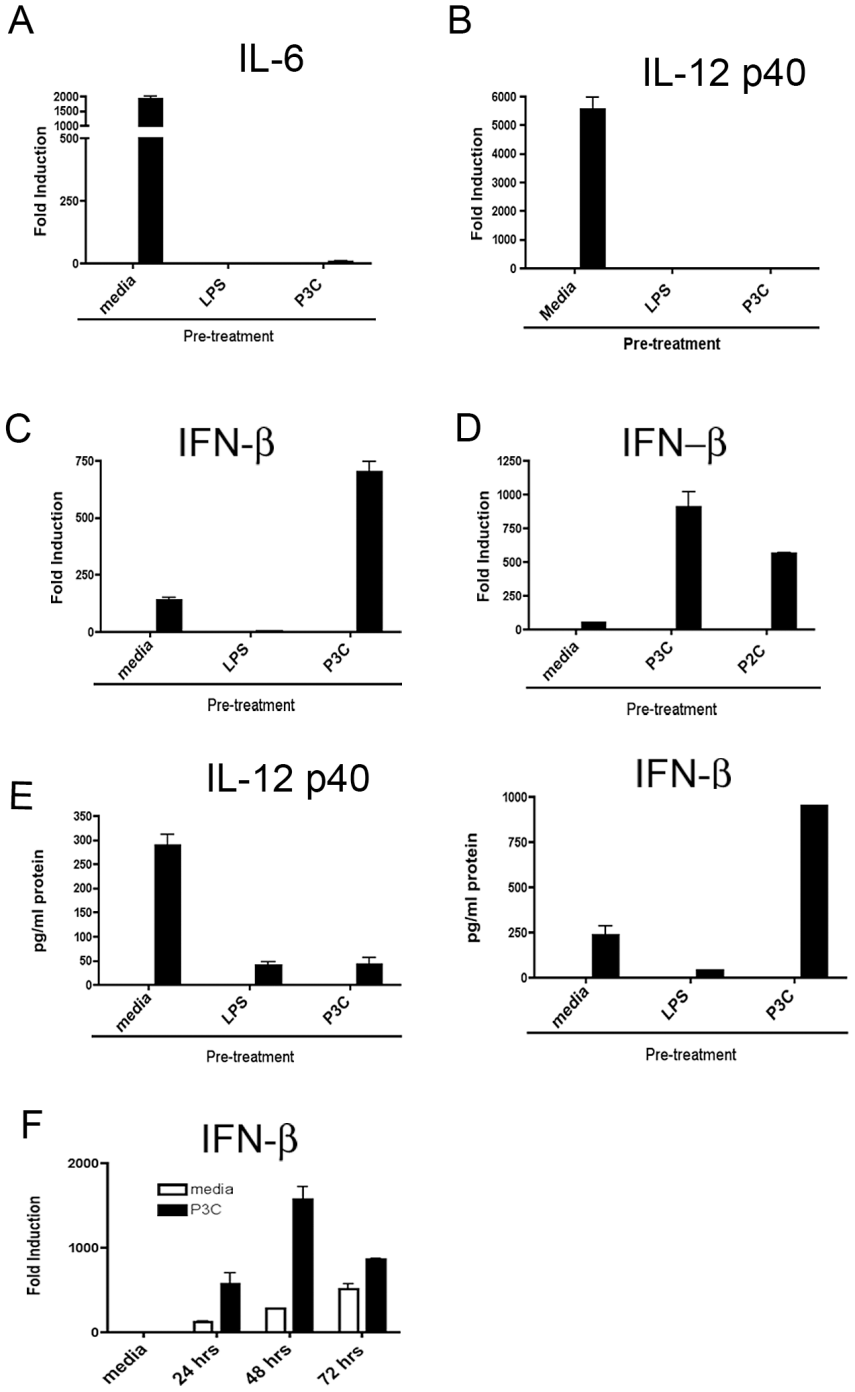
TLR2 ligands can selectively potentiate IFN-β production induced via TLR4. (A–C) Primary peritoneal macrophages were treated overnight with media or the indicated TLR ligands, and then re-stimulated with *E. coli* LPS (100 ng/ml) for 4 hrs (A and B) or 2 hrs (C) and gene expression for IL-6 (A), IL-12 p40 (B), or IFN-β (C) was analyzed by qRT-PCR. (D) Primary peritoneal macrophages were treated overnight with media or Pam3Cys or Pam2Cys (250 ng/ml) and re-stimulated after overnight incubation with *E. coli* LPS (100 ng/ml) for 2 hrs before extraction of RNA and analysis of gene expression. (E) Cell supernatants from macrophages treated as in (A–C) were analyzed for IL-12 p40 and IFN-β protein by ELISA. (F) Peritoneal macrophages were treated overnight with media or Pam3Cys (250 ng/ml) and re-stimulated either immediately with LPS for 2 hrs or following an additional 24, 48, or 72 hr resting period. Experiments were performed at least three times. Results from a single representative experiment are shown.

To characterize further the effects of TLR cross-talk on the induction of important inflammatory genes, primary mouse peritoneal macrophages were treated with media alone, or media supplemented with ligands for TLR 2 (P3C) or TLR4 (LPS). After overnight stimulation, the primary stimulus was removed and the cells washed extensively and allowed to rest for 60 minutes. The macrophage cultures were next re-stimulated with the TLR4 ligand, *E. coli* LPS, for 2 or 4 hrs and examined for gene induction by qRT-PCR. LPS induction of both the classical pro-inflammatory genes IL-6 and IL-12 p40 was strongly inhibited by prolonged TLR pre-stimulation (Figure 1, A and B). This is the predicted pattern described previously and known as “homotolerance” or “heterotolerance,” respectively [20]. Unexpectedly, however, when we examined the effect of TLR pre-stimulation on the LPS-mediated induction of type I interferon (IFN-β), we found the nature of the effect to be critically dependent on whether the initial stimulation had come through TLR2 or TLR4. Pretreatment with LPS (TLR4) sharply inhibited subsequent IFN-β induction in response to LPS (Figure 1C). However, pretreatment with P3C (TLR2) markedly potentiated IFN-β by nearly 10-fold at the mRNA level when compared to media-pretreated macrophages (Figure 1C). These TLR2-dependent changes in LPS-mediated gene induction were also observed at the level of protein (Figure 1 E). This pattern of enhanced IFN-β induction was also seen when induced by virally relevant ligands (forthcoming figures; to be presented later). Enhancement of subsequent IFN-β induction by pre-stimulation was independent of IFN signaling as the IFNAR^−/−^ macrophages exhibited a similar pattern of induction (Figure S2)). Additionally, pre-treating macrophages with a panel of other ligands relevant to innate immunity did not reproduce the priming effect seen with TLR2 ligands (Figure S1).

As a role for TLR2 in potentiating Type I interferon has not been described we sought to distinguish the molecular determinants of this effect. TLR2 functions as an obligate heterodimer with either TLR1 or TLR6, depending on the nature of the ligand [21]. We used ligands for TLR2/6 (P2C) or TLR2/1 (P3C) heterodimers in pretreatment experiments, and both potentiated subsequent IFN-β production in response to LPS via TLR4 (Figure 1D). The effect of TLR2 pre-stimulation on IFN-β was long-lived, lasting for at least 72 hrs following the removal of the pre-treatment TLR2 stimulus (Figure 1F). We also observed a requirement for a prolonged initial exposure (at least 8 h) to TLR2 ligands to elicit a measurable increase in IFN-β induction (Figure S3).

**Figure 2:**
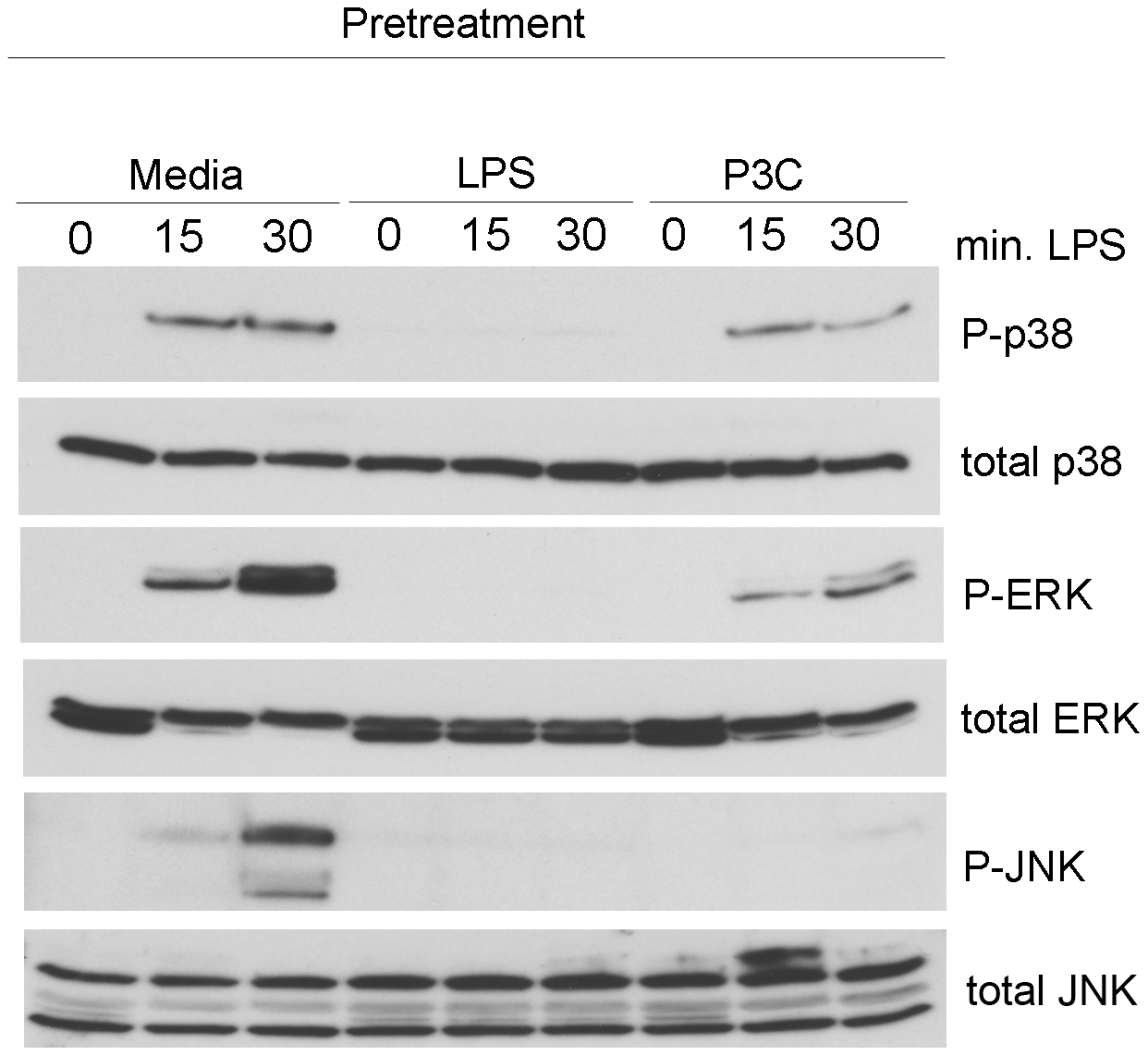
TLR2 pretreatment does not potentiate TLR4-driven MAPK activation. Primary peritoneal macrophages were treated overnight with media alone, 100 ng/ml *E. coli* LPS, or 250 ng/ml Pam3Cys. All cells were washed and then re-stimulated with 100 ng/ml E. coli LPS for 15 or 30 minutes. Cell lysates were analyzed by Western blotting using the indicated antibodies. Experiments were performed at least three times and the results of a representative experiment is shown.

The unexpected result that “priming” peritoneal macrophages with TLR2 ligands enhanced TLR4-dependent IFN-β mRNA expression led us to speculate that components of the signal transduction apparatus downstream of TLR4 were being augmented by TLR2 stimulation. To test this hypothesis, we initially profiled MAPK signaling in response to LPS in media-pretreated, LPS-pretreated, or P3C-pretreated macrophages. Stimulation of naïve macrophages with LPS resulted in robust activation of the MAPKs p38, ERK p42/p44, and JNK as evidenced by increased phosphorylation (Figure 2). Pretreating macrophages with LPS overnight completely ablated LPS-driven MAPK activation (Figure 2), a result consistent with previous reports on endotoxin tolerance [20], [22]. Pretreatment of macrophages with the TLR2 ligand P3C also reduced the activation of p38 and ERK, and almost completely inhibited activation of JNK by LPS (Figure 2). These data confirm prior reports that TLR2-mediated “heterotolerance” is less efficient than TLR4 “homotolerance” with respect to MAPK activation [20]. Based on these data, augmentation of MAPK signaling induced by TLR2-mediated “priming” cannot account for TLR2-mediated upregulation of IFN-β mRNA.

**Figure 3:**
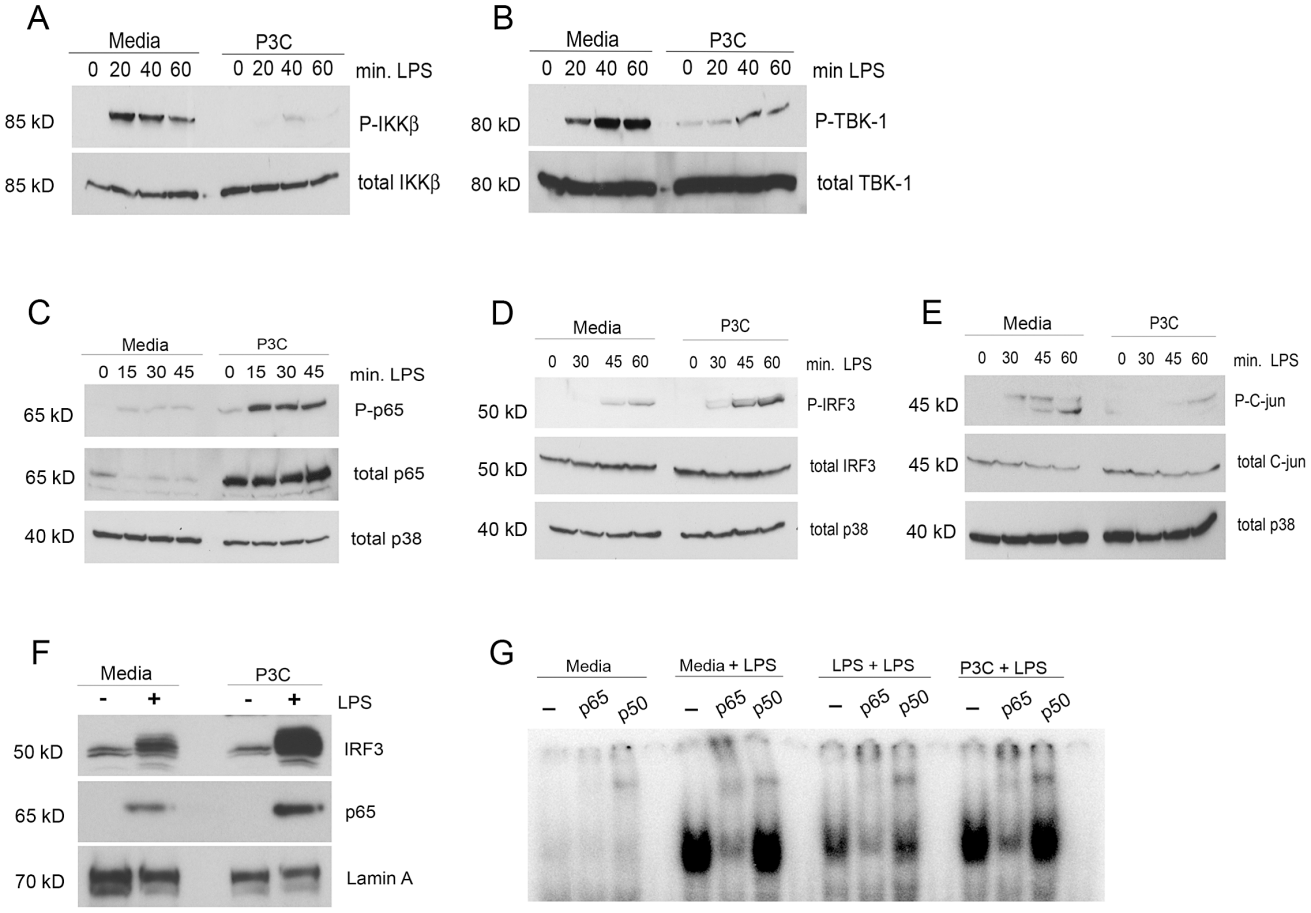
TLR2 selectively potentiates TLR4 driven transcription factor activation. (A) and (B) Primary peritoneal macrophages were treated overnight with media alone or with Pam3Cys (250 ng/ml) and then re-stimulated with *E. coli* LPS (100 ng/ml) for 0, 20, 40, or 60 min before harvesting cell lysates for Western analysis with indicated antibodies. (C–E) These experiments were carried out as in (A) with the indicated time-course of LPS re-stimulation. (F) Cells were treated overnight as in (A) and then re-stimulated for 60 min with LPS. Nuclear lysates were harvested and used for Western analysis with indicated antibodies. (G) Nuclear lysates from cells treated overnight as indicated and then re-stimulated with LPS were used in EMSA. Where indicated, antibodies were added to reaction to super-shift complexes. Experiments were performed at least three times and the results of a representative experiment are shown.

Two discrete signaling arms are initiated upon TLR4 signaling and contribute to IFN-β activity [20], [23], [24]. Classical NF-κB activation through the canonical kinase, IKKβ, results in phosphorylation of the transcription factor, p65, that subsequently contributes to induction of not only most proinflammatory cytokines and chemokines, but also IFN-β. Activation of the non-canonical kinase, TBK-1, via the TRIF/TRAM arm of the TLR4 signaling pathway leads to activation of transcription factor, IRF-3, that, together with NF-κB and other transcription factors, activates transcription of IFN-β and other similarly regulated genes. We examined IKKβ activation by phospho-specific Western analysis of medium-pretreated and P3C-primed macrophages. LPS induced strong IKKβ activation in naïve macrophages by 20 min that declined significantly by 60 min (Figure 3A). In marked contrast, LPS induced extremely weak IKKβ activation in the TLR2 (P3C)-pretreated cells that was detected transiently at 40 min (Figure 3A). LPS-mediated TBK-1 phosphorylation in TLR2-primed cells activity was reduced when compared to that achieved in naïve cells, but was not inhibited to the degree as IKKβ (Figure 3B). As neither IKKβ nor TBK-1 activity could account for the increase in IFN-β induction in TLR2-primed macrophages, we sought to examine the effect of TLR2 priming directly on key transcription factors known to be involved in regulating the IFN-β promoter [25]. We observed that phosphorylation of the NF-κB constituent p65 on residue serine 536 was dramatically enhanced in the P3C-pretreated cells when compared to the naïve macrophages (Figure 3C). Unexpectedly, we also observed a highly significant increase in the steady-state levels of total p65 in TLR2-primed cells that likely contributes to the increase in phosphorylated p65 (Figure 3C). Phosphorylation of the transcription factor IRF3 (on serine 396) was increased in P3C-primed macrophages; however, activation was not accompanied by increase in the levels of total IRF3 and, therefore, increased total IRF3 cannot explain the observed increase in phosphorylation (Figure 3D).

The transcription factor c-Jun is a constituent of the heterodimeric transcription factor, AP-1, that has also been shown to be essential for activation of IFN-β transcription [26]. In the case of TLR4, c-jun phosphorylation is also known to be downstream of MAPK signaling. In contrast to p65 and IRF3, LPS-driven phosphorylation of c-jun was significantly reduced in P3C-primed peritoneal macrophages (Figure 3E). This result is in agreement with the previous MAPK results showing a loss of JNK activation in P3C-pretreated macrophages (Figure 2). Both p65 and IRF3 are phosphorylated by their proximal kinases, IKKβ and TBK-1, respectively, while in the cytosol. Following phosphorylation, both p65 and IRF3 enter the nucleus where they are competent to bind to the IFN-β promoter. To examine whether the observed increased phosphorylation of p65 and IRF3 resulted in increased nuclear import, we performed nuclear fractionation of both medium-pretreated and P3C-primed macrophages after stimulation with medium only or with LPS for 60 min, followed by Western analysis for p65 and IRF3 (Figure 3F). We observed significantly augmented nuclear accumulation of both p65 and IRF3 in the P3C pre-treated cells when compared to the medium-pretreated, naïve macrophages (Figure 3F).

Since nuclear accumulation of p65 may not correlate directly with DNA binding, we performed an EMSA using our nuclear fractions from naïve, LPS-pretreated, or P3C-primed macrophages that were re-stimulated with media or LPS for 60 min (Figure 3G). Nuclear proteins from naïve (media-pretreated) macrophages exhibited strong binding to the NF-κB probe when stimulated with LPS (Figure 3G). The binding complex could be super-shifted with antibodies directed against either p65 or p50 (Figure 3G). LPS-tolerized macrophages re-stimulated with LPS showed diminished NF-κB activation, and the mobility of this complex could also be super-shifted by addition of either anti-p65 or anti-p50 antibodies. P3C-pretreated macrophages displayed NF-κB binding activity that was similar in intensity to that seen in LPS-stimulated, medium-pretreated macrophages.

**Figure 4:**
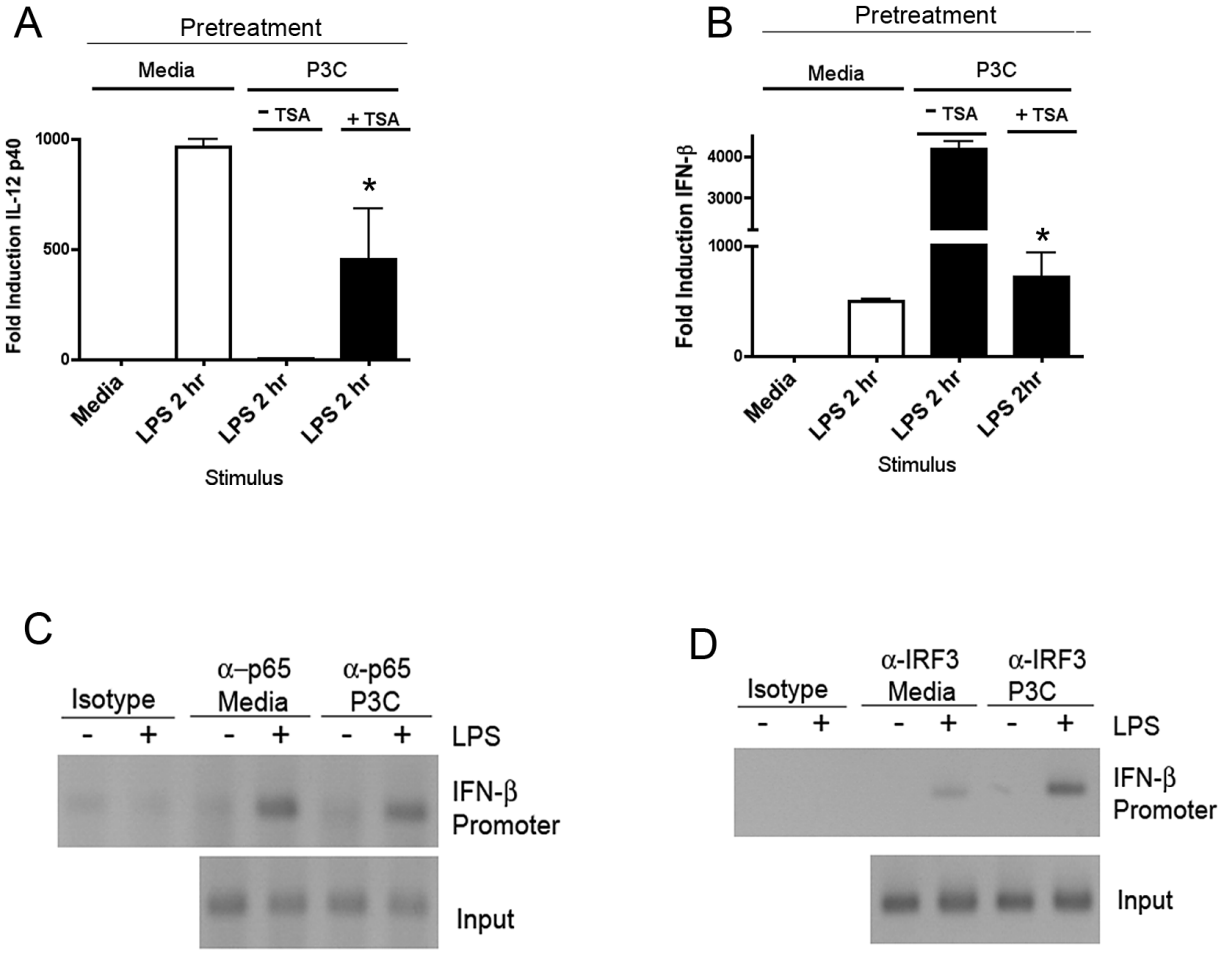
Enhanced transcription factor occupancy on the IFN-β promoter. (A) and (B). Primary peritoneal macrophages were treated for 8 hrs with media alone, or with 250 ng/ml Pam3Cys, or Pam3Cys plus 50 nM Trichostatin A (TSA). Following stimulation, cells were washed and allowed to rest overnight, at which time the cells were re-stimulated with 100 ng/ml *E. coli* LPS for 4 (A) or 2 (B) hrs. Gene expression was analyzed by qRT-PCR. (C) and (D) Macrophages treated overnight with media alone or 250 ng/ml Pam3Cys were re-stimulated with *E. coli* LPS (250 ng/ml) for 60 min and used in Chromatin Immuno-precipitation (ChIP) assays with antibodies against the indicated transcription factors. Precipitated DNA was amplified using primers directed against a region of the IFN-β promoter. Experiments were performed at least three times and the results were obtained from a single representative experiment.

A key question that arises from these observations is the following: why, as in the case of the P3C-primed macrophages, where NF-κB activity is not strongly diminished, do we not see an increase in the activity of all known NF-κB-responsive genes, *e.g.*, IL-6 or IL-12 p40 (Figures 1A and 1B)? One answer may lie in the works of Medzhitov et al. and McCall et al. who showed that in LPS homotolerance, some normally LPS-responsive genes are transcriptionally silenced through chromatin remodeling such that these promoters are no longer able to interact with transcription factors, regardless of the levels of activated transcription factors [27], [28]. Therefore, we hypothesized that if such a state also occurs in TLR2-induced heterotolerance, it may be possible to abrogate the inactive/tolerant phenotype by preventing remodeling during the initial (“tolerizing”) stimulus. To test this hypothesis, primary macrophages were treated with media alone, Pam3Cys, or Pam3Cys in the presence of Trichostatin A (TSA), a known inhibitor of histone deacetylase activity. Each group of cells was then re-stimulated with LPS as before and assayed for the induction of IL-12 p40 by qRT-PCR. As expected (Figure 1B), TLR2 priming of macrophages eliminated induction of IL-12 p40 mRNA by LPS (Figure 4A). Concurrent treatment of macrophages with TSA and TLR2 ligand significantly preserved the LPS inducibility of IL-12 p40 (Figure 4A). P3C-primed macrophages exhibited a significance increase in IFN-β mRNA induced by LPS that was reversed by the presence of TSA. Thus, manipulation of histone deacetylase activity reverses “heterotolerance.”

While the treatment with TSA strongly argues for a role for chromatin remodeling in TLR2 heterotolerance leading to diminished IL-12 p40 mRNA expression, treatment with chemical inhibitors does not directly assay for transcription factor binding. We therefore also performed chromatin immunoprecipitation (ChIP) analysis on medium- vs. P3C-pretreated macrophages (Figure 4, C and D). Following 60 min of LPS stimulation in naïve or P3C-primed cells, cross-linked chromatin was immunoprecipitated with antibody against either IRF3 or p65/RelA. PCR was used to amplify bound chromatin fragments corresponding to the IFN-β enhancer region. Consistent with our results with the EMSA, p65 binding activity was maintained in P3C-primed cells at levels seen in medium-pretreated macrophages. However, IRF3 binding to the IFN-β promoter was significantly enhanced in the P3C-primed cells Figure 4C and D). As an additional control, we assayed for the recruitment of RNA pol II to the IL-12p40 promoter following 60 minutes of LPS stimulation in naïve and TLR2 primed macrophages (Figure S4). TLR2 priming strongly inhibited RNA pol II recruitment to IL-12 p40, arguing for negative chromatin remodeling in the regulation of this promoter.

**Figure 5:**
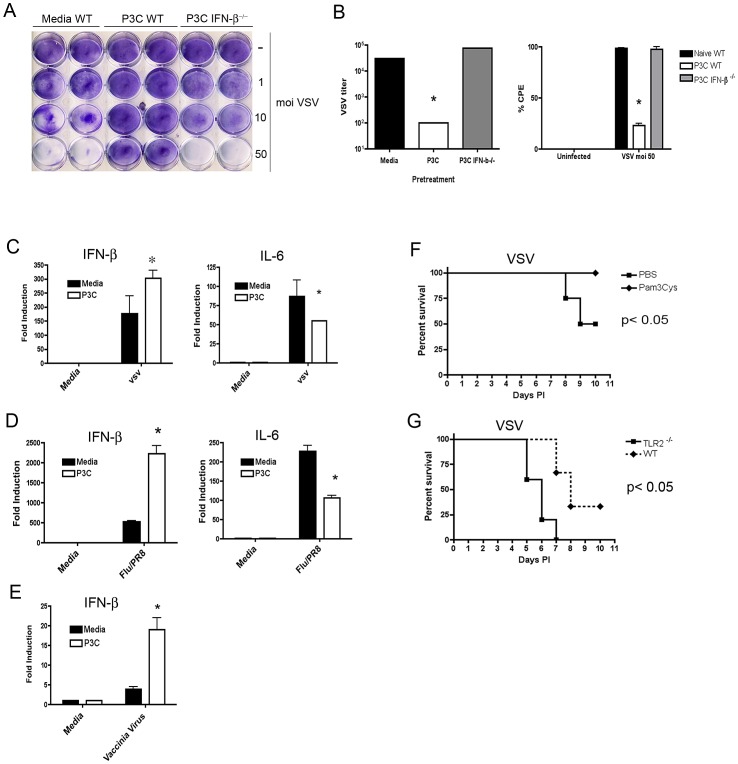
TLR2 ligation enhances *in vitro* and *in vivo* viral resistance and IFN-β production. (A) WT C57BL/6 or IFN-β^−/−^ primary peritoneal macrophages were treated overnight with media alone or with 250 ng/ml Pam3Cys and subsequently infected with the indicated MOI of Vesicular Stomatitis Virus. After 24 hrs of infection, adherent monolayers were stained with crystal violet. (B) (left panel) Supernatant from triplicate wells of macrophages infected with VSV (MOI = of 10) for 24 hours were harvested and viral titer was determined by plaque titration on BHK cells. (right panel) Trypan Blue was used to stain macrophages infected with VSV (MOI = 50) for 24 hrs. A minimum of 10 fields per well were counted. (C) Macrophages were pretreated with media alone or Pam3Cys for 24 hrs and subsequently infected with indicated MOI of VSV and harvested after six hrs for gene expression analysis. (D) Macrophages were pretreated with media alone or Pam3Cys for 24 hrs and subsequently infected with influenza strain PR8 at an MOI = 1 for 6 hrs and harvested for gene expression analysis. (E) Macrophages were pretreated with media alone or with Pam3Cys for 24 hrs and subsequently infected with Vaccinia Virus (WR strain) at an MOI of 5 for 8 hours prior to harvesting for gene expression analysis (F) WT 6–8 week female BALBc/J mice were injected i.p. with 0.5 ml saline or 100 µg Pam3Cys in saline 24 hrs prior to i.n. infection with 1×10^6^ pfu VSV. N = 12 mice per treatment group (G) WT or TLR2^−/−^ mice were infected i.n with 1×10^7^ pfu of VSV in saline. N = 12 mice per group. All experiments were performed at least three times and data from a representative experiment is shown for each.

Having narrowed the likely mechanism for TLR2 priming of Type I interferon to increased transcriptional activity, we interrogated the larger biological significance of this phenomena. Given that the primary function of IFN-β is in antiviral defense, we next examined the effect of TLR2 priming on the course of a model virus infection. To this end, WT primary peritoneal macrophages were infected *in vitro* with Vesicular Stomatitis Virus (VSV Indiana Strain) at increasing MOI, without or with prior P3C priming. Additionally, we primed IFN-β^−/−^ macrophages with TLR2 ligand and these cells were similarly infected with VSV. Significant inhibition of VSV-induced cytopathic effect (CPE) was observed in cells pretreated with P3C (Figure 5A). The protective capacity of TLR2 priming was dependent on having an intact IFN-β gene (Figure 5A) as evidenced by the failure of P3C to protect IFN-β^−/−^ macrophages. TLR2 priming inhibited viral growth, rather than simply inducing cell death, as plaque titration of VSV showed a >2 log inhibition (Figure 5B, left panel). Quantitation of CPE by measuring the crystal violet stain eluted from cells remaining bound to the plate strongly paralleled quantification of viral replication obtained by plaque assay (Figure 5B, right panel). We next sought to determine whether VSV infection of P3C-primed cells elicited greater production of IFN-β mRNA than seen in naïve cells. To this end, naïve or P3C-primed macrophages were infected with VSV, and IFN-β mRNA was measured by qRT-PCR 6 hours post-infection. As predicted, VSV infection induced greater levels of IFN-β mRNA after pre-stimulation (Figure 5C). Interestingly, TLR2 priming resulted in diminished IL-6 mRNA following infection (Figure 5C). To extend our virus studies further, naïve and primed macrophages were infected with a second RNA virus, influenza. The mouse adapted influenza strain, PR8, also elicited greater production of IFN-β in P3C-primed cells as well as diminished production of IL-6 mRNA (Figure 5D). As both VSV and influenza are relatively small RNA viruses, we also utilized Vaccinia Virus (WR strain) as a model DNA virus. We observed enhanced IFN-β induction by Vaccinia Virus at 8 hours post-infection, although we were not able to detect IL-6 at this time point (Figure 5E).

The significant protection afforded by P3C priming *in vitro* encouraged us to extend our virus studies *in vivo*. Female BALB/c mice were administered 500 µl of PBS or PBS containing 100 µg Pam3Cys i.p. Twenty-four hrs later, mice were infected intranasally (i.n.) with 1×10^6^ pfu VSV. Mice were monitored for mortality and morbidity for up to 10 days post-infection. We observed complete protection from a VSV LD_50_ in mice that received a single priming dose of P3C (Figure 5F; p<0.05). Similar experiments were carried out in which medium- or P3C-pretreated mice were challenged with ∼5000 TCID_50_ influenza PR8 (∼LD_100_). P3C-primed mice exhibited a delayed mean time to death upon PR8 challenge compared to unprimed, infected mice (data not shown). To examine the contribution of TLR2 to viral defense in the absence of prophylactic P3C administration, WT or TLR2^−/−^ mice were infected i.n. with a lethal dose of VSV. Loss of TLR rendered animals more susceptible to fatal VSV infection (Figure 5G).

**Figure 6:**
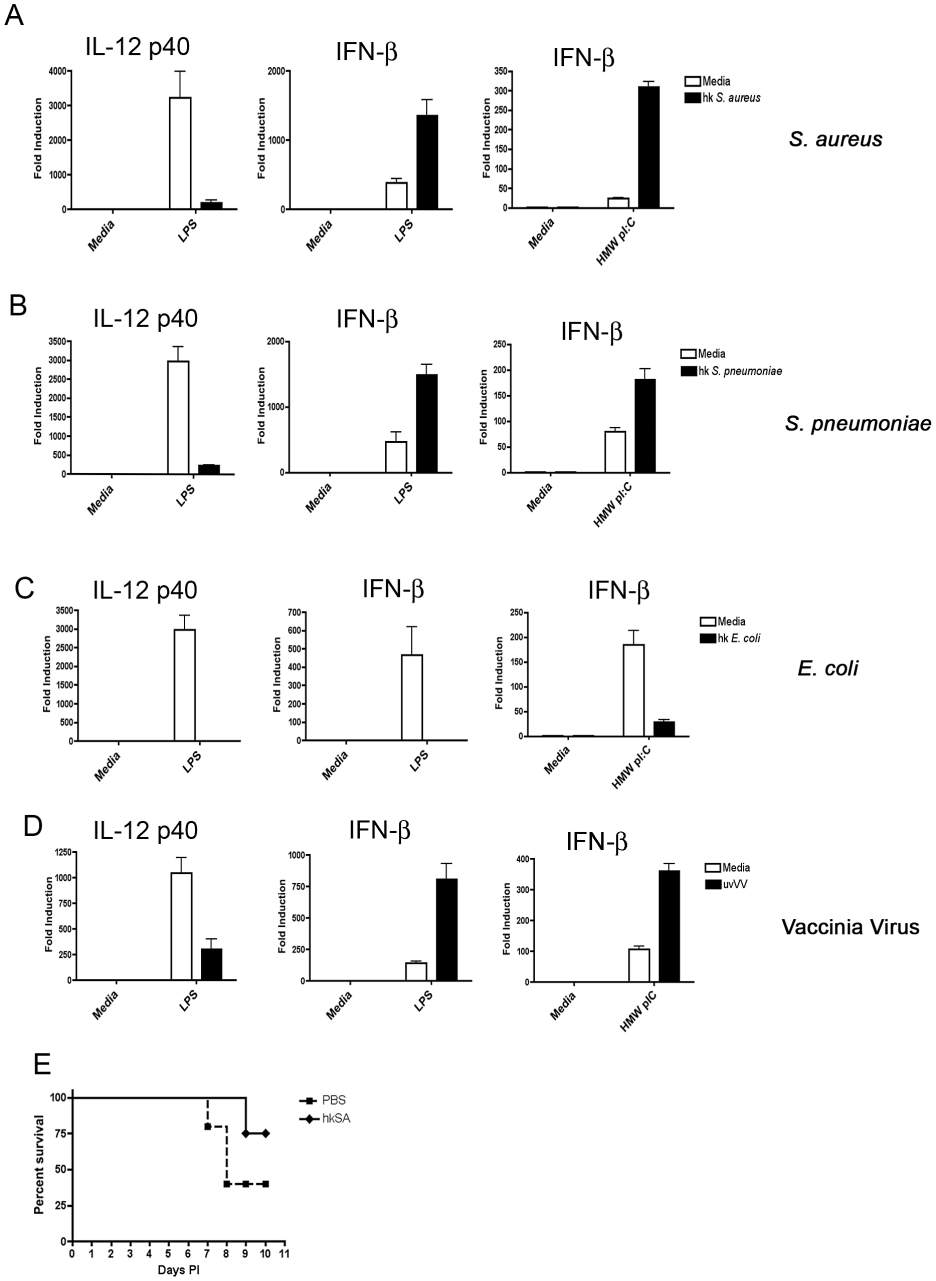
Gram positive bacteria and select viruses, but not Gram negative bacteria, can prime for TLR- and RLR-driven IFN-β production. (A–C) Primary peritoneal macrophages were incubated overnight with media alone or with the indicated heat killed bacteria (moi = 1). Following treatment, macrophages were washed 3X with PBS and re-stimulated either with *E coli* LPS (100 ng/ml) for 2 hours, or transfected with poly I:C (1 µg/ml) for 6 hours. (D) Macrophages were incubated with media or UV inactivated Vaccinia Virus (moi = 0.5) overnight and re-stimulated with E. coli LPS (100 ng/ml) for 2 hours or transfected with poly I:C for 6 hours. (E) BALB/cJ mice were injected i.p. with saline or heat-killed *S. aureus* 24 hours prior to i.n. infection with x10^7^ pfu of VSV. N = 12 mice per group. Experiments were performed three times with a representative experiment shown for each.

While it is clear that TLR2 can contribute to the regulation of IFN-β and innate antiviral immunity, our previous experiments utilized a pure, synthetic ligand, Pam3Cys, to establish TLR2 priming. Whether such priming could be accomplished by biological organisms in the context of infection has not been shown. To address this issue, peritoneal macrophages were incubated with heat-killed Gram positive bacteria *Staphylococcus aureus* overnight at an MOI of 1. Macrophages pre-incubated with *S. aureus* were subsequently stimulated with *E. coli* LPS or transfected with poly I:C to simulate a viral infection. Macrophages exposed to *S. aureus* displayed strongly ablated induction of IL-12 p40 and enhanced induction of IFN-β in response to LPS (Figure 6A). Similarly, transfected poly I:C induced far greater IFN-β in bacterially primed cells (Figure 6B). We confirmed these observations with a second clinically relevant Gram positive bacteria, *S. pneumoniae* (Figure 6B). Our initial experiments demonstrating divergent effects for TLR2 and TLR4 on subsequent type I IFN induction (Figure 1C) led us to speculate that a priming effect for IFN-β might be a common effect of Gram positive, but not Gram negative bacteria. To test this hypothesis, peritoneal macrophages were pre-incubated with media alone or heat-killed *E. coli* and then stimulated either with LPS or by transfected poly I:C. In marked contrast to Gram positive bacteria, pre-treatment with *E. coli* strongly suppressed IFN-β mRNA induced by both LPS and transfected poly I:C (Figure 6C). In addition to bacteria, some viruses are known to signal through TLR2, including Vaccinia Virus [13]. We assayed the ability of UV-inactivated Vaccinia Virus to regulate IFN-β production by LPS and transfected poly I:C. Vaccinia Virus was capable of effective priming for both TLR4 and the RLRs (Figure 6D). To demonstrate that *in vivo* priming by one organism might influence an infection by a second, we injected BALB/cJ mice i.p. with PBS or heat-killed *S. aureus* 24 hours prior to i.n. infection with VSV. We observed a significant protective effect induced by *S. aureus* against VSV infection (Figure 6E).

**Figure 7:**
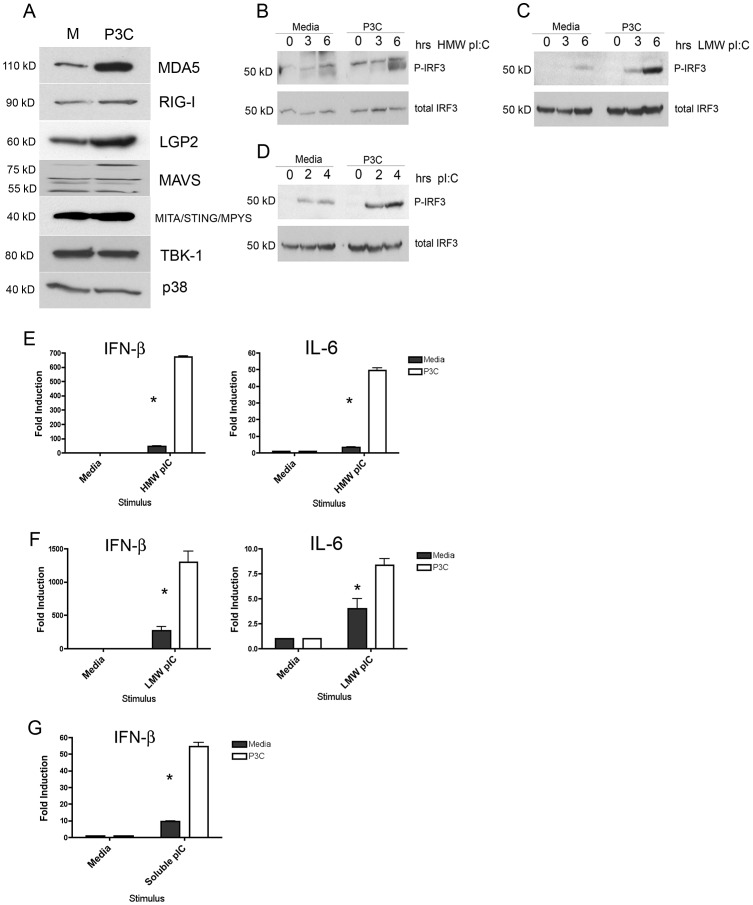
TLR2 enhances RLR expression and signaling. (A) Primary peritoneal macrophages were treated overnight with media alone or 250 ng/ml Pam3Cys and were analyzed by Western blot for expression of RLR family members. (B–D) Macrophages treated with media or Pam3Cys overnight were transfected with HMW poly I:C, (B) LMW poly I:C, (C) or non-transfected extracellular poly I:C (D) and analyzed for IRF3 activation by Western blot. (E) Cells treated as in (B) were used to examine gene expression by qRT-PCR. (F) Cells treated as in (C) were used to examine gene expression by qRT-PCR. (G) Cells treated as in (D) were used to examine gene expression by qRT-PCR. Experiments were performed three times with a representative experiment shown for each.

While it is clear that both VSV and influenza infections can involve TLR4 [29], [30], the cytosolic nucleic acid innate immune receptors play a large role in detecting RNA viruses and initiating an IFN response via the TBK-1/IRF3 axis. It was therefore important to determine if the antiviral effects of TLR2 priming are limited to the TLRs, and specifically TLR4, or whether IFN-β induction is potentiated by TLR2 priming through other families of cytosolic pattern recognition receptors. To this end, we examined the expression levels of the known cytosolic RNA sensors MDA5, RIG-I, and MAVS in medium- and P3C-treated macrophages. We observed a striking up-regulation in the expression of MDA5 protein following 24 hrs of P3C stimulation (Figure 7A). We also observed a much more modest, but reproducible, increase in RIG-I protein levels. As seen previously [31], MAVS appears as several bands of differing electrophoretic mobilities, of which only one appears to be increased in P3C-treated macrophages. The increase in abundance of these cytosolic RNA sensors led us to speculate that there may be a functional increase in sensitivity of this system in P3C-treated macrophages. Therefore, naïve and P3C-treated primary macrophages were transfected with High Molecular Weight (HMW) and Low Molecular Weight (LMW) poly I:C to stimulate MDA5 and RIG-I, respectively. As a control, we additionally treated control and P3C-pretreated macrophages with “free” poly I:C to stimulate TLR3. We examined a time course of IRF3 activation by Western analysis in each case (Figure 7, B–D) and IFN-β mRNA induction by qRT-PCR (Figures 7 E–G). HMW and LMW poly I:C, as well as soluble poly I:C, elicited dramatically greater activation of IRF3 in P3C-primed cells, correlating in each case with a strong increase in induction of IFN-β at the mRNA level. Interestingly, in the cases of transfected poly I:C, we did not observe an inhibition of IL-6 induction in P3C-primed cells.

**Figure 8:**
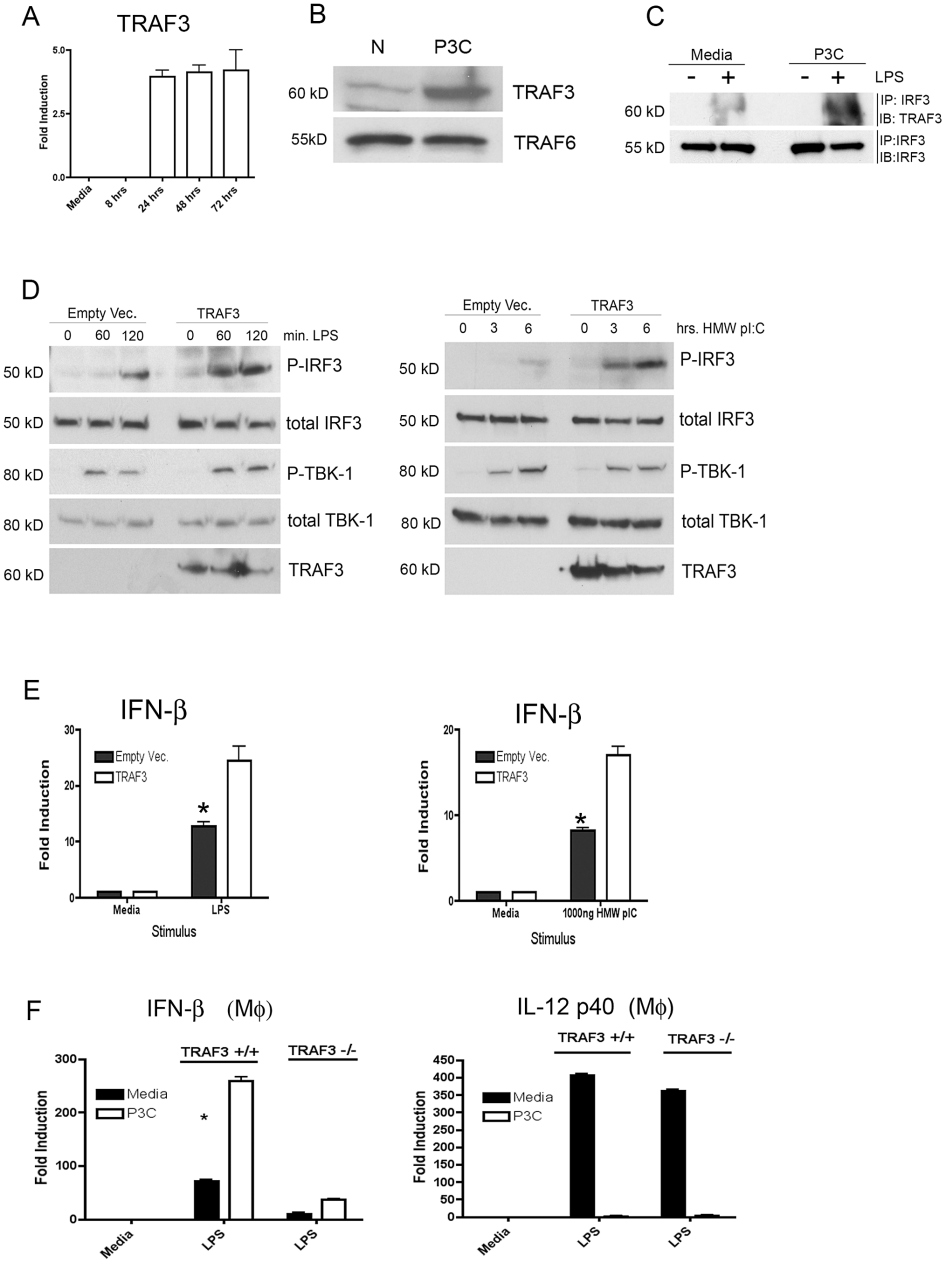
TRAF3 expression is elevated by TLR2 priming and over-expression is sufficient to potentiate TLR- and RLR-mediated IRF3 activation. (A) Primary peritoneal macrophages treated overnight with media alone or 250 ng/ml Pam3Cys were washed 3X with PBS and were harvested at the indicated times and analyzed by qRT-PCR for TRAF3 expression. (B) Macrophages were treated overnight with Pam3Cys (250 ng/ml) and analyzed by Western blot for TRAF3 expression. (C) Peritoneal macrophages were treated overnight with media or Pam3Cys and re-stimulated for 45 min with LPS (300 ng/ml). Cell lysates were immunoprecipitated with antibodies against IRF3 and blotted with antibody against TRAF3. (D and E, left panels) MAT4 HeLa cells transiently transfected with human TRAF3 were stimulated with *E. coli* LPS (300 ng/ml) for the times indicated and used in Western blot (D) or qRT-PCR (E). (D and E, right panels) MAT4 HeLa cells transiently transfected with human TRAF3 were transfected with Poly I:C and used in Western blotting (D) or qRT-PCR (E). (F) Peritoneal macrophages carrying a TRAF3 floxed gene and expressing CRE recombinase, or TRAF3-sufficient littermate control macrophages, were primed as in (A) and assayed for IFN-β induction or IL-12 p40 induction. All experiments were performed at least three times and data from a representative experiment is shown for each.

In seeking a common signaling element that might account for the potentiation in IFN-β induction by both TLRs and RLRs, we postulated that the ubiquitin ligase TRAF3 would be a strong candidate to mediate this effect. TRAF3 is immediately upstream of IRF3 activation in both the TLR and RLR pathways and TRAF3-null immune cells are defective in IFN induction via TLRs [32]–[34]. Initially, we profiled TRAF3 mRNA over a time course of Pam3Cys treatment and found a late increase in TRAF3 consistent with the time of IFN-β potentiation (Figure 8A). We examined the levels of TRAF3 and TRAF6 in the naïve and P3C-pretreated macrophages by Western analysis. Dramatically, we found that protein levels of TRAF3, but not TRAF6, were significantly higher in P3C-primed cells (Figure 8B). As a control, we tested additional innate immune ligands, and found that while TLR3 and TLR4 were capable of inducing TRAF3 mRNA to limited extents, the effect was significantly weaker than for TLR2 ligands (Figure S5). To ascertain whether elevated levels TRAF3 might lead to enhanced signaling complex formation, we performed co-immunoprecipitations from macrophages following 45 min of LPS treatment utilizing an antibody against IRF3 and blotting for co-precipitating TRAF3. We observed significantly greater amounts of TRAF3 in complex with IRF3 in TLR2-primed cells (Figure 8C). While TRAF3 is an essential component of TRIF signaling to IFN-β, it is not known whether an increase in TRAF3 by itself can potentiate TLR4-dependent IFN-β induction and IRF-3 activation. To test this hypothesis, we over-expressed TRAF3 in MAT4 cells, HeLa cells that constitutively express TLR4, stimulated them with LPS, and examined TBK-1 and IRF3 activation. TRAF3 over-expression significantly increased both signaling leading to P-IRF3 (Figure 8D, left panel) and IFN-β gene induction (Figure 8E, left panel). Over-expression of TRAF3 also increased IRF3 activation and IFN-β gene induction induced by transfection of cells with HMW poly I:C (Figures 8D and 8E, left panels respectively). Overexpression of TRAF3 did not significantly affect LPS-driven p65 phosphorylation (data not shown). To complement our over-expression studies, we performed TLR2 priming and LPS re-stimulation studies using littermate control and Lys-Cre TRAF3^flox/flox^ (TRAF3^−/−^) peritoneal macrophages. Loss of TRAF3 severely compromised LPS-driven IFN-β induction irrespective of prior TLR2 priming (Figure 8F, left panel). The overall enhancement of LPS-dependent IFN-β induction due to priming in TRAF3^+/+^ macrophages was 4.2-fold compared to unprimed cells. In contrast, TLR2 priming only enhanced IFN-β induction by an average of 1.7-fold in TRAF3^−/−^ macrophages. The loss of TRAF3 had little effect on the induction of IL-12 p40 (Figure 8F, right panel), thus illustrating the specificity of TRAF3 for the interferon inducing pathway.

In conclusion, we have identified a novel mechanism that accounts for the ability of TLR2 to increase antiviral immunity despite an inability to increase IFN levels directly. TLR2 priming of macrophages results in increased TRAF3 levels that lead to the selective priming of IFN-β production by multiple innate immune surveillance pathways and increased viral resistance.

## Discussion

The distinct subcellular localizations and the overlapping ligand chemistries among the receptors in the TLR and RLR families underscores the complementarity between these systems in providing a comprehensive surveillance network against viral infection. While often regarded as a receptor for bacterial lipoproteins, a critical role for TLR2 in antiviral defense has been established in several systems. The human viral pathogens Cytomeglovirus (CMV), Respiratory Syncytial Virus (RSV) and Varicella Zoster all have been shown to either directly encode TLR2 ligands among their structural proteins, or to require functional TLR2 for immune clearance *in vivo* [14], [16], [35]. Of particular interest for our present study, splenic levels of IFN-β are diminished in TLR2^−/−^ mice infected with mouse CMV [15]. A recent report has extended the connection between TLR2 and the innate response to virus [13]. In this work, using Vaccinia Virus as a model, an as yet unknown ligand on UV-inactivated Vaccinia was shown to be a potent TLR2 agonist. Unexpectedly, while Vaccinia could trigger TLR2-dependent cytokines from all TLR2-expressing cell types tested, it also selectively induced type I IFN in a TLR2-dependent manner, from Ly6C^hi^ “inflammatory monocytes.” However, the molecular mechanism underlying this phenomenon has yet to be fully elucidated. Our current work also provides evidence that UV-inactivated Vaccinia is a potent TLR2 agonist, and we propose that TLR2-based “priming” may be an additional mechanism by which TLR2 contributes to type I IFN production in tissues where innate immune cells other than inflammatory monocytes predominate. Such priming might work systemically during virus infection as free viral proteins capable of acting as TLR2 ligands are shed into the circulation from an infected tissue and travel to uninfected sites and encounter TLR2-expressing innate immune cells. However, it is important to note that a TLR2 priming-based mechanism need not only be of significance during infection with a single viral pathogen. We have demonstrated that TLR- and RLR-mediated IFN-β production is potently enhanced by TLR2 ligands derived from both Gram positive bacteria as well as virus. This could play a role in the pathogenesis of polymicrobial co-infections involving both a bacterial and a viral component. Indeed, synergistic IFN production has already been demonstrated *in vivo* in a model involving co-infection of *Streptococcus pneumonia* and influenza A in the upper airways of mice [36]. In this model, Gram positive bacterial infection enhances viral induced IFN-β, that in turn, enhances bacterial growth [36].

Recent literature also suggests that TLR-RLR cross-talk with respect to IFN production may not only occur under dynamic conditions of infection and inflammation. Steady-state priming of innate IFN production in macrophages by unknown components of the intestinal microbiota has been definitively demonstrated [37]. Our data also support the notion that prior ligation of TLR2 leads to a reduction in the subsequent induction of classical pro-inflammatory cytokines such as IL-12 and IL-6, while simultaneously increasing IFN-β responsiveness. In fact, a dynamic balance between IL-12 and IFN-β was recently suggested and shown to depend mechanistically on the levels of IRF transcription factor activation [19]. Whether such a mechanism is operative in our experimental systems remains to be determined.

Our data strongly supports the hypothesis that this potentiation likely occurs, in part, via a TLR-dependent amplification of the levels of the K63 ubiquitin ligase TRAF3. TRAF3 is uniquely positioned at a common node in the IFN-inducing pathways downstream of both TLRs and RLRs, and our data clearly show that TLR2 pre-stimulation greatly increases the amount of IRF3 activation for a given dose of challenge ligand. Precisely how, on an inter-molecular level, this priming affect occurs remains to be elucidated. This question is made all the more interesting, given that the positive effects of TRAF3 in our system take place in a context in which we see diminished TBK-1 activation downstream of TLR4 (Fig. 3B). The elegant studies by Oganesyan et al. [33] and Hacker et al. [32] support a model in which in response to TLR4 ligation, TRAF3 is part of a multi-protein complex that contains both TBK-1 and IRF3 and in which TRAF3 is clearly required for IRF3 activation. Since over-expression of TRAF3 alone is not sufficient to initiate signaling to the IFN-β promoter ([33] and Figure 8D), we propose that the principal effect of elevated TRAF3 levels is to dramatically enhance the kinetics by which TBK-1 can interact with and phosphorylate IRF3. Such a schema would be logical given the enzymatic activity of TRAF3 as a K63 ubiquitin ligase capable of assisting in the assembly of multi-protein complexes. This scenario is the first of which we are aware, in which TRAF3 levels are used as a rheostatic mechanism by which the innate immune response is tuned. The potential cell- and tissue-type specificity of the effects of TLR2 priming, and by extension, TRAF3, in reshaping the transcriptional responses to innate immune receptor ligation remains an area for future investigation. This is particularly relevant given that in contrast to macrophages and dendritic cells, TRAF3^−/−^ B cells exhibit increased responses to TLR ligands [38], [39]. Additionally, TRAF3 has a well-established role as a negative regulator of the non-cannonical NF-κB pathway in B cells [40] that may provide greater complexity to the crosstalk between TLR2 and other pathways such as those downstream of CD40. It should be noted that the presence of residual priming effects of TLR2 ligands in the TRAF3^−/−^ macrophages (Figure 8 F) indicates that additional TLR2-responsive factors beyond TRAF3 contribute to this phenomena and need to be further investigated. For example, one possible additional factor that might contribute to enhanced IFN-β induction could be the cytosolic receptor MDA5, as steady-state levels of MDA5 protein are dramatically up-regulated following TLR2 priming (Figure 7A).

In addition to TRAF3, we found that levels of the NF-κB family member, p65, were greatly enhanced following TLR2 priming. Such TLR-dependent regulation of p65 has not been previously reported, but is analogous to the accumulation of RelB reported in endotoxin tolerance [41]. The molecular mechanism of this p65 regulation remains to be elucidated, but may represent an exciting new area of governance for NF-κB responses. The enhanced accumulation of p65 protein levels may be important in maintaining NF-κB activation downstream of TLR4 in situations of reduced IKKβ kinase complex activity such as is found in TLR2-primed cells (Figure 3A).

It should also be noted that the present work has several important implications with respect to our understanding of macrophage TLR tolerance. Tolerance in macrophages as a result of prolonged exposure to ligands for TLRs has long been seen as a means to limit excessive acute inflammation in cases of disseminated infection. At the same time, not all TLR-responsive genes are “tolerizable” [42], although the mechanism for the discrimination between tolerized and non-tolerized genes is far from clear. Our work suggests that a macrophage made “tolerant” by exposure to TLR2 ligands may, in fact, selectively prime some classes of promoters, such as those responsive to IRF3, by up-regulating certain downstream signaling nodes (*e.g.*, TRAF3) and in this way render the host hyper-vigilant to viral infection.

## Materials and Methods

### Ethics statement

Animal work performed for this study complied with all applicable provisions of the Animal Welfare Act, the U.S. Government Principles for the Utilization and Care of Vertebrate Animals Used in Testing, Research, and Training, the Public Health Services (PHS) Policy on the Humane Care and Use of Laboratory Animals and the Guide for the Care and Use of Laboratory Animals (8th Ed.). The IACUC protocol governing this work was reviewed by the IACUC committee of the University of Maryland Baltimore School of Medicine. IACUC protocol A3200-01. This committee specifically approved the protocol for this work.

### Cell lines and mice

Primary murine peritoneal macrophages were prepared as described previously [20]. Briefly, 3 ml of 3% sterile fluid thioglycollate (Remel) was injected i.p. into 6–8 wk old, wild-type (WT) C57BL/6J mice or BALB/cJ (Jackson Laboratories, Bar Harbor, ME). Four days later, macrophages were harvested by peritoneal lavage with sterile saline. IFN-β-null mice (IFN-β^−/−^) and MyD88-null mice (MyD88^−/−^), backcrossed onto a C57BL/6J background (N≥8), were bred in-house as described previously [43]. IFNAR^−/−^ mice and control littermates were a kind gift of Dr. Matthew Frieman (University of Maryland Baltimore). TLR2 null mice (TLR2^−/−^) were a kind gift of Dr. Rose Viscardi (University of Maryland Baltimore). TRAF3^−/−^ and littermate control MEFs were a kind gift of Dr. Genhong Cheng (UCLA Medical School) and were maintained in DMEM (BioWhittaker) supplemented with 10% FBS, 2 mM L-glutamine, 100 U/ml penicillin, and 100 mg/ml streptomycin. Lys-Cre TRAF3^flox/flox^ macrophages were generated by Dr. Ping Xie (Rutgers University). HEK293T (ATCC, Manassas, VA) cells were cultured in DMEM (BioWhittaker) supplemented with 10% FBS, 2 mM L-glutamine, 100 U/ml penicillin, and 100 mg/ml streptomycin. The RAW 264.7 macrophage-like cell line (ATCC) was cultured in RPMI 1640 (BioWhittaker) supplemented with 10% FBS, 2 mM L-glutamine, 100 U/ml penicillin, and 100 mg/ml streptomycin. MAT4 cells (a kind gift of Dr. Liwu Li, Virginia Tech University, Blacksburg, VA) were maintained in DMEM supplemented as with 293T cells.

### Antibodies and reagents

Antibodies against phospho-IRF-3 (Serine 396), total IRF-3, phospho-TBK-1 (Serine 172), total TBK-1, phospho-IKKβ (Serine 176/180), total IKKβ, phospho-ERK1/2 (Thr202/Tyr 204), total ERK1/2, phospho-p38 (Thr 180/Tyr 182), total p38, phospho-JNK (Thr 183/Tyr 185), total JNK, phospho-c-jun (Serine 63), total c-jun and phospho-p65 (Serine 536), MDA5, MAVS, RIG-I, and STING were obtained from Cell Signaling (Danvers, MA). Antibody against p50 was purchased from Millipore (Bellirica, MA). Polyclonal antibodies against total p65, C-rel, as well as monoclonal anti TRAF3 were from Santa Cruz Biotechnology (Santa Cruz, CA). Polyclonal anti-TRAF3 antisera was obtained from IMGENEX. Anti DHX58/LGP2 was obtained from Abnova (Taipei City, Taiwan). Protein-free, phenol/water-extracted *Escherichia coli* K235 LPS was prepared as described elsewhere [44]. S-[2,3-Bis(palmitoyloxy)-(2-RS)-propyl]-N-palmitoyl-(R)-Cys-Ser-Lys_4_-OH (P3C), R848, CpG, poly dA:T, recombinant *Salmonella typhimurium* flagellin, and transfection reagent-conjugated high molecular weight (HMW) and low molecular weight (LMW) polyinosinic:polycytidylic acid (poly I:C) were purchased from Invivogen (San Diego, CA). Trichostatin A (TSA) and MG132 were obtained from CalBiochem (Carlsbad, CA). Heat killed S. aureus and S. pneumonia were purchased from Invivogen (San Diego, CA). Strain DH5α *E.coli* was obtained from Invitrogen (Grand Island, NY) and were heat killed by incubation at 60°C for one hour prior to use.

### Transfection of HeLa cells

MAT4 cells were plated at a density of 5×10^5^ cells per well of a six well dish and transfected 24 hrs later with 1 µg of empty vector, or vector that expresses human TRAF3 using Superfect (Qiagen) according to manufacturer's recommended protocol. Twenty-four hr after transfection, cells were stimulated with media alone or media containing 100 ng/ml LPS or transfected with poly I:C. Cells were then washed 3× in PBS and whole cell lysates harvested.

### Western blot analysis

Whole cell lysates from primary murine macrophages or MAT4 HeLa cells were obtained by the addition of lysis buffer (20 mM HEPES, 1.0% Triton X-100, 0.1% SDS, 150 mM NaCl, 10 mM NaF, 1 mM PMSF) and subsequent incubation at 4°C. Cell lysates were separated by electrophoresis in a denaturing SDS-PAGE gel, and subsequent transfer to PVDF membrane. Blots were incubated overnight in relevant primary antibodies at 4°C, washed 3X with PBS, and then incubated with appropriate HRP-conjugated, secondary antibody (Jackson Immunochemicals, West Grove, PA). Blots were developed following incubation in ECL Plus Western Blotting Detection Reagent (Amersham Bioscience, Piscataway, NJ).

### Viral infections

Vesicular Stomatitis Virus (Indiana Strain; ATCC) was grown and titered as previously described [45]. For infection of primary macrophages, macrophages were washed 1X with PBS and infected at the indicated multiplicity of infection (MOI) in serum-free RPMI for 1 hr at 37°C with occasional rocking. Infection media was removed and cells were cultured for an additional 24–48 hrs in RPMI containing 2% FBS. For in vivo infection, BALB/c mice were first anaesthetized with isofluorane and subsequently infected intranasally (i.n.) with virus resuspended in PBS as described [43].

Influenza strain A/PR/8/34 (“PR8”) was obtained from the ATCC and propagated as described previously [46] and was the kind gift of Dr. Donna Farber (Columbia University, NY). *In vitro* infections were conducted as described above for VSV. Vaccinia Virus (Western Reserve) was obtained from the NIH AIDS Research and Reference Reagent Program, Division of AIDS, NIAID, NIH: Vaccinia WR from Dr Bernard Moss. In vitro Vaccinia infections were performed as descried for VSV. UV inactivation of vaccinia was carried out using a StrataLinker (Stratagene corp.) at a dose of 5 J/cm^2^.

### Preparation of nuclear extracts and EMSA

Nuclear extracts were prepared using a nuclear extraction kit (Active Motif, Carlsbad, CA) according to the manufacturer's instructions. The NF-κB consensus oligonucleotide, 5′-agttgaggggactttcccaggc-3′, from the murine IgκB light chain gene enhancer was synthesized by the Biopolymer and Genomics Core Laboratory (University of Maryland, Baltimore, MD). DNA probes were ^32^P end-labeled with T4 polynucleotide kinase (Invitrogen Life Technologies), as recommended by the manufacturer. EMSA was conducted as described previously [47].

### Quantitative Real Time PCR (qRT-PCR)

Total mRNA was isolated from peritoneal macrophages using TRIZOL (Invitrogen Carlsbad, CA) reagent according to manufacturer's instructions. A total of 1 µg of RNA was utilized in oligo(dT) cDNA synthesis (Promega RT system A3500). qRT-PCR was carried out using an ABI Prism 7900HT Sequence Detection System (Applied Biosystems) utilizing SYBR Green Reagent (Applied Biosystems) and transcript-specific primers. mRNA expression profiles were normalized to levels of the housekeeping gene hypoxanthine-guanine phosphoribosyltransferase (HPRT) in each sample and the fold change in expression was calculated by the 2^−ΔΔCt^ method [48]

### Chromatin Immunoprecipitation assays

ChIP assays were carried out using the Active Motif (Carlsbad CA) ChIP-IT Express Enzymatic kit according to manufacturers instructions.

## Supporting Information

Figure S1 **Priming of TLR4 induced IFN-β by ligands of the innate immune system.** Primary peritoneal macrophages were treated overnight either with media alone or with Pam3Cys (250 ng/ml), soluble poly I:C (50 mg/ml), *E. coli* LPS (25 ng/ml), recombinant *Salmonella* flagellin (100 ng/ml), transfected poly I:C (500 ng/ml), or transfected poly dA:dT (500 ng/ml). Cells were washed extensively and re-stimulated with *E. coli* LPS (25 ng/ml) for two hours. Total RNA was harvested and used to analyze IFN-β expression by qRT-PCR.
(TIF)

Figure S2 **TLR2 priming of TLR4 induced IFN-β is type I Interferon receptor-independent.** Primary peritoneal macrophages harvested from C57BL/6J WT and IFNAR^−/−^ mice were treated overnight with media alone or with Pam3Cys (250 ng/ml) and subsequently re-stimulated with *E. coli* LPS (25 ng/ml) for two hours. Total RNA was harvested and used to analyze IFN-βby qRT-PCR.
(TIF)

Figure S3 **TLR2 priming of TLR4 induced IFN-β requires prolonged exposure to TLR2 ligands.** Primary peritoneal macrophages were treated for the indicated times with media alone or with Pam3Cys (250 ng/ml) and subsequently re-stimulated with *E. coli* LPS (25 ng/ml) for two hours. Total RNA was harvested and used to analyze IFN-βby qRT-PCR.
(TIF)

Figure S4 **Loss of RNA pol II recruitment to the IL-12 p40 promoter in TLR2 primed macrophages.** Primary peritoneal macrophages were treated overnight either with media alone, or with Pam3Cys (250 ng/ml). Cells were washed extensively and re-stimulated with *E. coli* LPS (100 ng/ml) for 60 min. Cell lysates were used in Chromatin Immuno-precipitation (ChIP) with a monoclonal antibody directed against RNA pol II. Precipitated DNA was amplified using primers against a region of the IL-12 p40 promoter.
(TIF)

Figure S5 **Differential TRAF3 induction by ligands of the innate immune system.** Primary peritoneal macrophages were treated for 8 hours either with media alone or with Pam3Cys (250 ng/ml), soluble poly I:C (50 mg/ml), *E. coli* LPS (20 ng/ml), recombinant *Salmonella* flagellin (100 ng/ml), transfected poly I:C (500 ng/ml), or transfected poly dA:dT (500 ng/ml). Total RNA was harvested and used to analyze TRAF3 expression by qRT-PCR.
(TIF)

## References

[1] Yoneyama M, Kikuchi M, Matsumoto K, Imaizumi T, Miyagishi M, et al (2005) Shared and unique functions of the DExD/H-box helicases RIG-I, MDA5, and LGP2 in antiviral innate immunity. J Immunol 175: 2851–2858. 16116171 10.4049/jimmunol.175.5.2851

[2] Kato H, Takeuchi O, Sato S, Yoneyama M, Yamamoto M, et al (2006) Differential roles of MDA5 and RIG-I helicases in the recognition of RNA viruses. Nature 441: 101–105. 16625202 10.1038/nature04734

[3] Loo YM, Fornek J, Crochet N, Bajwa G, Perwitasari O, et al (2008) Distinct RIG-I and MDA5 signaling by RNA viruses in innate immunity. J Virol 82: 335–345. 17942531 10.1128/JVI.01080-07 PMC2224404

[4] Zhang Z, Kim T, Bao M, Facchinetti V, Jung SY, et al (2011) DDX1, DDX21, and DHX36 helicases form a complex with the adaptor molecule TRIF to sense dsRNA in dendritic cells. Immunity 34: 866–878. 21703541 10.1016/j.immuni.2011.03.027 PMC3652560

[5] Sharma S, Fitzgerald KA (2011) Innate immune sensing of DNA. PLoS Pathog 7: e1001310. 21533068 10.1371/journal.ppat.1001310 PMC3080846

[6] Stetson DB, Medzhitov R (2006) Recognition of cytosolic DNA activates an IRF3-dependent innate immune response. Immunity 24: 93–103. 16413926 10.1016/j.immuni.2005.12.003

[7] Brennan K, Bowie AG (2010) Activation of host pattern recognition receptors by viruses. Curr Opin Microbiol 13: 503–507. 20538506 10.1016/j.mib.2010.05.007

[8] Finberg RW, Kurt-Jones EA (2004) Viruses and Toll-like receptors. Microbes Infect 6: 1356–1360. 15596120 10.1016/j.micinf.2004.08.013

[9] Liu SY, Sanchez DJ, Cheng G (2011) New developments in the induction and antiviral effectors of type I interferon. Curr Opin Immunol 23: 57–64. 21123041 10.1016/j.coi.2010.11.003 PMC3822007

[10] Schoggins JW, Wilson SJ, Panis M, Murphy MY, Jones CT, et al (2011) A diverse range of gene products are effectors of the type I interferon antiviral response. Nature 472: 481–485. 21478870 10.1038/nature09907 PMC3409588

[11] Gonzalez-Navajas JM, Lee J, David M, Raz E (2012) Immunomodulatory functions of type I interferons. Nat Rev Immunol 12: 125–135. 22222875 10.1038/nri3133 PMC3727154

[12] Kawai T, Akira S (2011) Toll-like receptors and their crosstalk with other innate receptors in infection and immunity. Immunity 34: 637–650. 21616434 10.1016/j.immuni.2011.05.006

[13] Barbalat R, Lau L, Locksley RM, Barton GM (2009) Toll-like receptor 2 on inflammatory monocytes induces type I interferon in response to viral but not bacterial ligands. Nat Immunol 10: 1200–1207. 19801985 10.1038/ni.1792 PMC2821672

[14] Murawski MR, Bowen GN, Cerny AM, Anderson LJ, Haynes LM, et al (2009) Respiratory syncytial virus activates innate immunity through Toll-like receptor 2. J Virol 83: 1492–1500. 19019963 10.1128/JVI.00671-08 PMC2620898

[15] Szomolanyi-Tsuda E, Liang X, Welsh RM, Kurt-Jones EA, Finberg RW (2006) Role for TLR2 in NK cell-mediated control of murine cytomegalovirus in vivo. J Virol 80: 4286–4291. 16611887 10.1128/JVI.80.9.4286-4291.2006 PMC1472014

[16] Wang JP, Kurt-Jones EA, Shin OS, Manchak MD, Levin MJ, et al (2005) Varicella-zoster virus activates inflammatory cytokines in human monocytes and macrophages via Toll-like receptor 2. J Virol 79: 12658–12666. 16188968 10.1128/JVI.79.20.12658-12666.2005 PMC1235827

[17] Nish S, Medzhitov R (2011) Host defense pathways: role of redundancy and compensation in infectious disease phenotypes. Immunity 34: 629–636. 21616433 10.1016/j.immuni.2011.05.009 PMC3143490

[18] Bakaletz LO (2004) Developing animal models for polymicrobial diseases. Nat Rev Microbiol 2: 552–568. 15197391 10.1038/nrmicro928 PMC7097426

[19] Negishi H, Yanai H, Nakajima A, Koshiba R, Atarashi K, et al (2012) Cross-interference of RLR and TLR signaling pathways modulates antibacterial T cell responses. Nat Immunol 13: 659–666. 22610141 10.1038/ni.2307

[20] Dobrovolskaia MA, Medvedev AE, Thomas KE, Cuesta N, Toshchakov V, et al (2003) Induction of in vitro reprogramming by Toll-like receptor (TLR)2 and TLR4 agonists in murine macrophages: effects of TLR “homotolerance” versus “heterotolerance” on NF-kappa B signaling pathway components. J Immunol 170: 508–519. 12496438 10.4049/jimmunol.170.1.508

[21] Kang JY, Lee JO (2011) Structural biology of the Toll-like receptor family. Annu Rev Biochem 80: 917–941. 21548780 10.1146/annurev-biochem-052909-141507

[22] Medvedev AE, Sabroe I, Hasday JD, Vogel SN (2006) Tolerance to microbial TLR ligands: molecular mechanisms and relevance to disease. J Endotoxin Res 12: 133–150. 16719986 10.1179/096805106X102255

[23] Piao W, Song C, Chen H, Diaz MA, Wahl LM, et al (2009) Endotoxin tolerance dysregulates MyD88- and Toll/IL-1R domain-containing adapter inducing IFN-beta-dependent pathways and increases expression of negative regulators of TLR signaling. J Leukoc Biol 86: 863–875. 19656901 10.1189/jlb.0309189 PMC2796624

[24] Yamamoto M, Sato S, Mori K, Hoshino K, Takeuchi O, et al (2002) Cutting edge: a novel Toll/IL-1 receptor domain-containing adapter that preferentially activates the IFN-beta promoter in the Toll-like receptor signaling. J Immunol 169: 6668–6672. 12471095 10.4049/jimmunol.169.12.6668

[25] Wathelet MG, Lin CH, Parekh BS, Ronco LV, Howley PM, et al (1998) Virus infection induces the assembly of coordinately activated transcription factors on the IFN-beta enhancer in vivo. Mol Cell 1: 507–518. 9660935 10.1016/s1097-2765(00)80051-9

[26] Falvo JV, Parekh BS, Lin CH, Fraenkel E, Maniatis T (2000) Assembly of a functional beta interferon enhanceosome is dependent on ATF-2-c-jun heterodimer orientation. Mol Cell Biol 20: 4814–4825. 10848607 10.1128/mcb.20.13.4814-4825.2000 PMC85927

[27] Foster SL, Hargreaves DC, Medzhitov R (2007) Gene-specific control of inflammation by TLR-induced chromatin modifications. Nature 447: 972–978. 17538624 10.1038/nature05836

[28] Yoza BK, McCall CE (2011) Facultative heterochromatin formation at the IL-1 beta promoter in LPS tolerance and sepsis. Cytokine 53: 145–152. 21078560 10.1016/j.cyto.2010.10.007 PMC3021647

[29] Shinya K, Ito M, Makino A, Tanaka M, Miyake K, et al (2012) The TLR4-TRIF pathway protects against H5N1 influenza virus infection. J Virol 86: 19–24. 22031950 10.1128/JVI.06168-11 PMC3255869

[30] Stout RD, Suttles J (2005) Immunosenescence and macrophage functional plasticity: dysregulation of macrophage function by age-associated microenvironmental changes. Immunol Rev 205: 60–71. 15882345 10.1111/j.0105-2896.2005.00260.x PMC1201508

[31] Seth RB, Sun L, Ea CK, Chen ZJ (2005) Identification and characterization of MAVS, a mitochondrial antiviral signaling protein that activates NF-kappaB and IRF 3. Cell 122: 669–682. 16125763 10.1016/j.cell.2005.08.012

[32] Hacker H, Redecke V, Blagoev B, Kratchmarova I, Hsu LC, et al (2006) Specificity in Toll-like receptor signalling through distinct effector functions of TRAF3 and TRAF6. Nature 439: 204–207. 16306937 10.1038/nature04369

[33] Oganesyan G, Saha SK, Guo B, He JQ, Shahangian A, et al (2006) Critical role of TRAF3 in the Toll-like receptor-dependent and -independent antiviral response. Nature 439: 208–211. 16306936 10.1038/nature04374

[34] Takeshita F, Tanaka T, Matsuda T, Tozuka M, Kobiyama K, et al (2006) Toll-like receptor adaptor molecules enhance DNA-raised adaptive immune responses against influenza and tumors through activation of innate immunity. J Virol 80: 6218–6224. 16775309 10.1128/JVI.00121-06 PMC1488967

[35] Compton T, Kurt-Jones EA, Boehme KW, Belko J, Latz E, et al (2003) Human cytomegalovirus activates inflammatory cytokine responses via CD14 and Toll-like receptor 2. J Virol 77: 4588–4596. 12663765 10.1128/JVI.77.8.4588-4596.2003 PMC152130

[36] Nakamura S, Davis KM, Weiser JN (2011) Synergistic stimulation of type I interferons during influenza virus coinfection promotes Streptococcus pneumoniae colonization in mice. J Clin Invest 121: 3657–3665. 21841308 10.1172/JCI57762 PMC3163966

[37] Abt MC, Osborne LC, Monticelli LA, Doering TA, Alenghat T, et al (2012) Commensal bacteria calibrate the activation threshold of innate antiviral immunity. Immunity 37: 158–170. 22705104 10.1016/j.immuni.2012.04.011 PMC3679670

[38] Hildebrand JM, Yi Z, Buchta CM, Poovassery J, Stunz LL, et al (2011) Roles of tumor necrosis factor receptor associated factor 3 (TRAF3) and TRAF5 in immune cell functions. Immunol Rev 244: 55–74. 22017431 10.1111/j.1600-065X.2011.01055.x PMC3202299

[39] Xie P, Poovassery J, Stunz LL, Smith SM, Schultz ML, et al (2011) Enhanced Toll-like receptor (TLR) responses of TNFR-associated factor 3 (TRAF3)-deficient B lymphocytes. J Leukoc Biol 90: 1149–1157. 21971520 10.1189/jlb.0111044 PMC3236554

[40] Xie P, Stunz LL, Larison KD, Yang B, Bishop GA (2007) Tumor necrosis factor receptor-associated factor 3 is a critical regulator of B cell homeostasis in secondary lymphoid organs. Immunity 27: 253–267. 17723217 10.1016/j.immuni.2007.07.012 PMC2084086

[41] Yoza BK, Hu JY, Cousart SL, Forrest LM, McCall CE (2006) Induction of RelB participates in endotoxin tolerance. J Immunol 177: 4080–4085. 16951372 10.4049/jimmunol.177.6.4080

[42] Henricson BE, Manthey CL, Perera PY, Hamilton TA, Vogel SN (1993) Dissociation of lipopolysaccharide (LPS)-inducible gene expression in murine macrophages pretreated with smooth LPS versus monophosphoryl lipid A. Infect Immun 61: 2325–2333. 8388859 10.1128/iai.61.6.2325-2333.1993 PMC280852

[43] Shirey KA, Nhu QM, Yim KC, Roberts ZJ, Teijaro JR, et al (2011) The anti-tumor agent, 5,6-dimethylxanthenone-4-acetic acid (DMXAA), induces IFN-beta-mediated antiviral activity in vitro and in vivo. J Leukoc Biol 89: 351–357. 21084628 10.1189/jlb.0410216 PMC3040469

[44] McIntire FC, Sievert HW, Barlow GH, Finley RA, Lee AY (1967) Chemical, physical, biological properties of a lipopolysaccharide from Escherichia coli K-235. Biochemistry 6: 2363–2372. 4867999 10.1021/bi00860a011

[45] Vogel SN, Friedman RM, Hogan MM (2001) Measurement of antiviral activity induced by interferons alpha, beta, and gamma. Curr Protoc Immunol Chapter 6: Unit 6 9. 10.1002/0471142735.im0609s37 18432822

[46] Palladino G, Mozdzanowska K, Washko G, Gerhard W (1995) Virus-neutralizing antibodies of immunoglobulin G (IgG) but not of IgM or IgA isotypes can cure influenza virus pneumonia in SCID mice. J Virol 69: 2075–2081. 7884853 10.1128/jvi.69.4.2075-2081.1995 PMC188873

[47] Polumuri SK, Toshchakov VY, Vogel SN (2007) Role of phosphatidylinositol-3 kinase in transcriptional regulation of TLR-induced IL-12 and IL-10 by Fc gamma receptor ligation in murine macrophages. J Immunol 179: 236–246. 17579043 10.4049/jimmunol.179.1.236

[48] Livak KJ, Schmittgen TD (2001) Analysis of relative gene expression data using real-time quantitative PCR and the 2(-Delta Delta C(T)) Method. Methods 25: 402–408. 11846609 10.1006/meth.2001.1262

