# Supplementary figures and images for: Reprogramming of Murine Macrophages through TLR2 Confers Viral Resistance via TRAF3-Mediated, Enhanced Interferon Production
Source: PLoS Pathog. 2013 Jul 11;9(7):e1003479. doi: 10.1371/journal.ppat.1003479 (PMC3708851; doi:10.1371/journal.ppat.1003479)

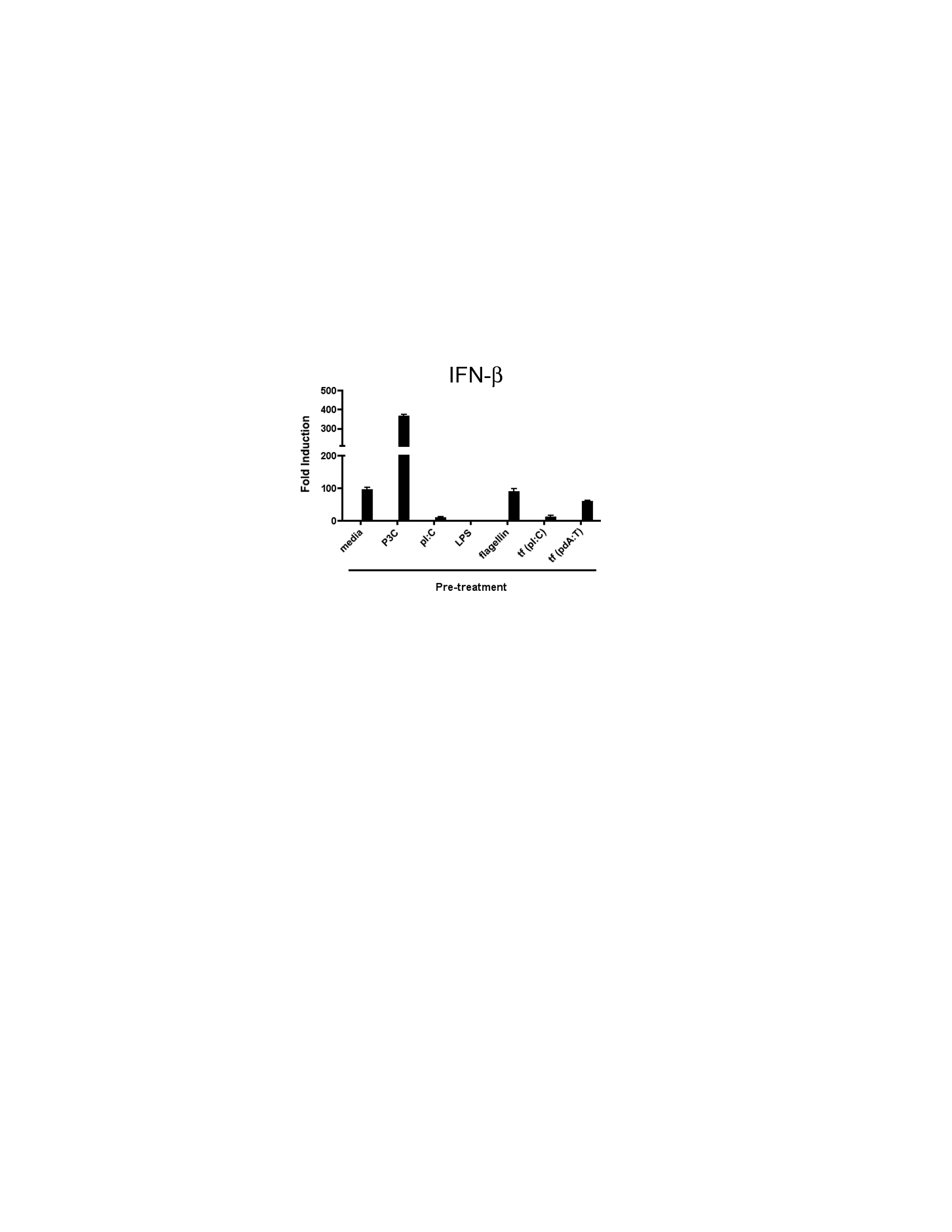

Supplement: Figure S1 — Priming of TLR4 induced IFN-β by ligands of the innate immune system. Primary peritoneal macrophages were treated overnight either with media alone or with Pam3Cys (250 ng/ml), soluble poly I:C (50 mg/ml), E. coli LPS (25 ng/ml), recombinant Salmonella flagellin (100 ng/ml), transfected poly I:C (500 ng/ml), or transfected poly dA:dT (500 ng/ml). Cells were washed extensively and re-stimulated with E. coli LPS (25 ng/ml) for two hours. Total RNA was harvested and used to analyze IFN-β expression by qRT-PCR. (TIF) [file ppat.1003479.s001.tif]

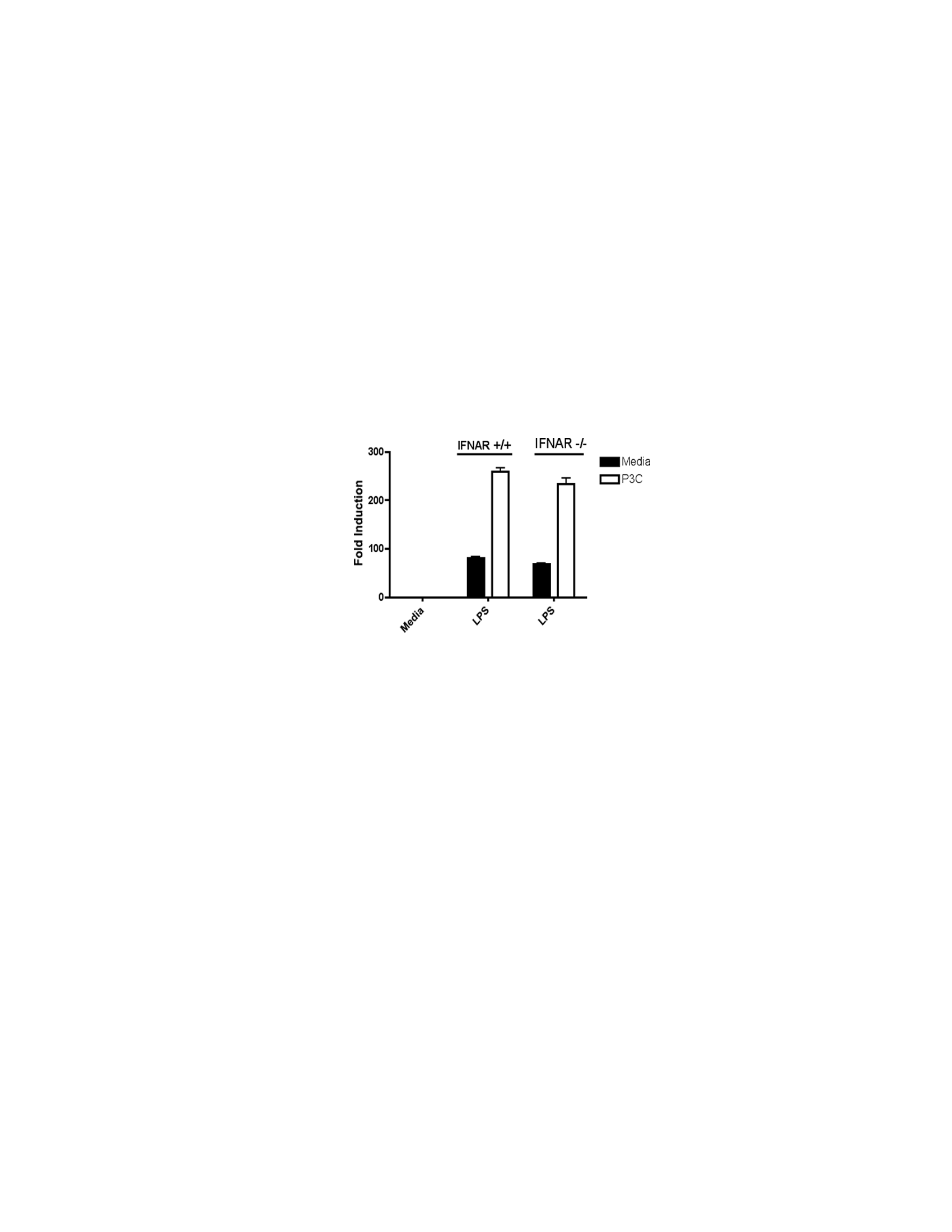

Supplement: Figure S2 — TLR2 priming of TLR4 induced IFN-β is type I Interferon receptor-independent. Primary peritoneal macrophages harvested from C57BL/6J WT and IFNAR−/− mice were treated overnight with media alone or with Pam3Cys (250 ng/ml) and subsequently re-stimulated with E. coli LPS (25 ng/ml) for two hours. Total RNA was harvested and used to analyze IFN-βby qRT-PCR. (TIF) [file ppat.1003479.s002.tif]

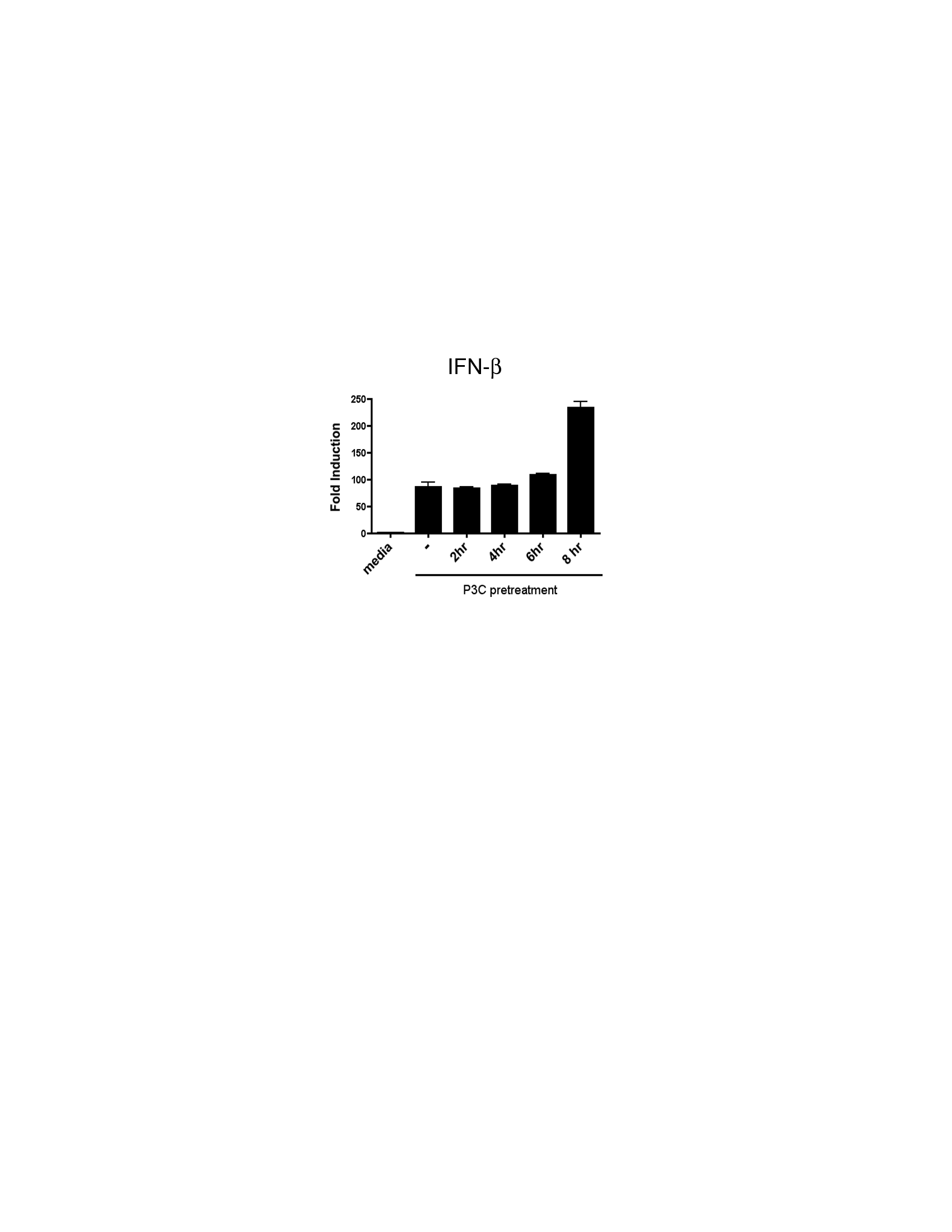

Supplement: Figure S3 — TLR2 priming of TLR4 induced IFN-β requires prolonged exposure to TLR2 ligands. Primary peritoneal macrophages were treated for the indicated times with media alone or with Pam3Cys (250 ng/ml) and subsequently re-stimulated with E. coli LPS (25 ng/ml) for two hours. Total RNA was harvested and used to analyze IFN-βby qRT-PCR. (TIF) [file ppat.1003479.s003.tif]

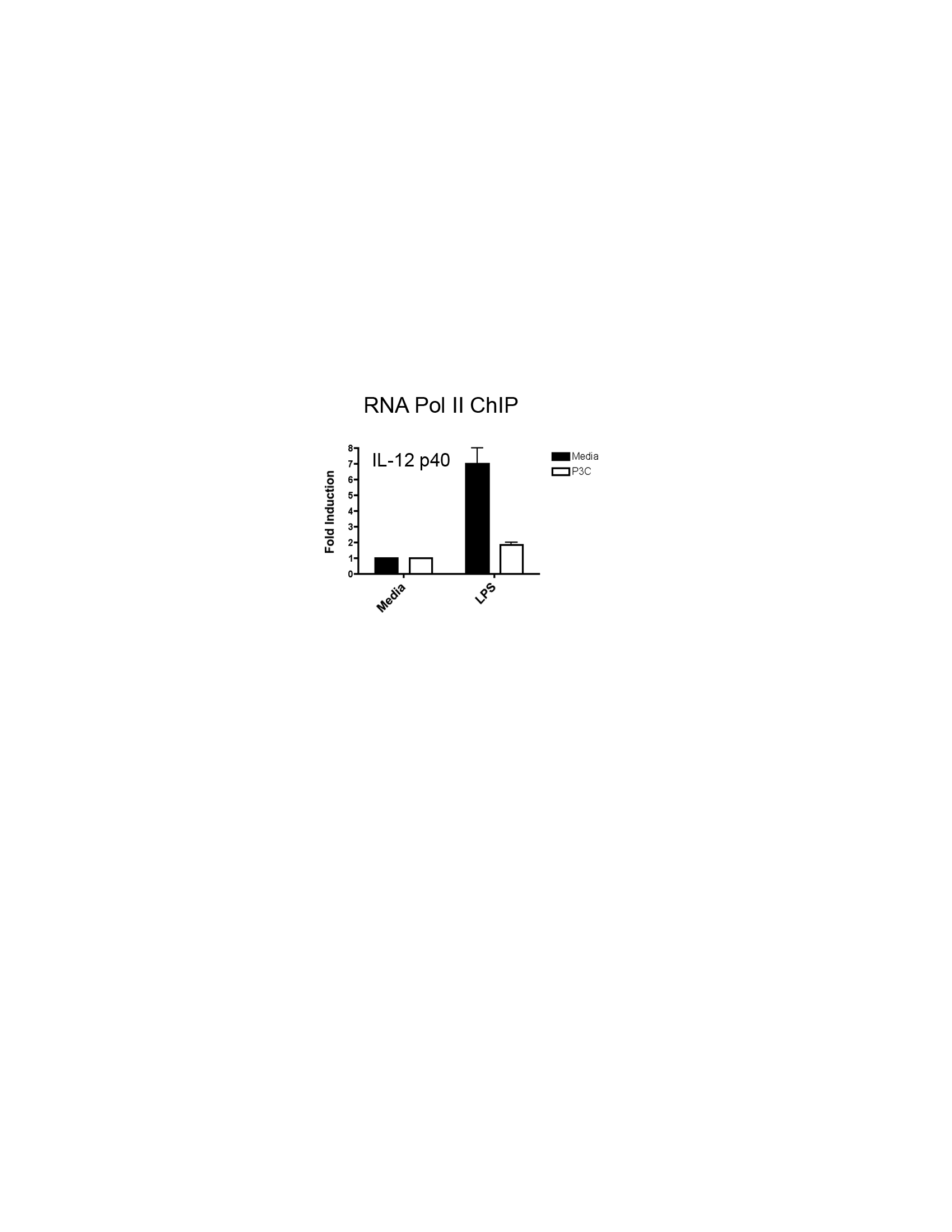

Supplement: Figure S4 — Loss of RNA pol II recruitment to the IL-12 p40 promoter in TLR2 primed macrophages. Primary peritoneal macrophages were treated overnight either with media alone, or with Pam3Cys (250 ng/ml). Cells were washed extensively and re-stimulated with E. coli LPS (100 ng/ml) for 60 min. Cell lysates were used in Chromatin Immuno-precipitation (ChIP) with a monoclonal antibody directed against RNA pol II. Precipitated DNA was amplified using primers against a region of the IL-12 p40 promoter. (TIF) [file ppat.1003479.s004.tif]

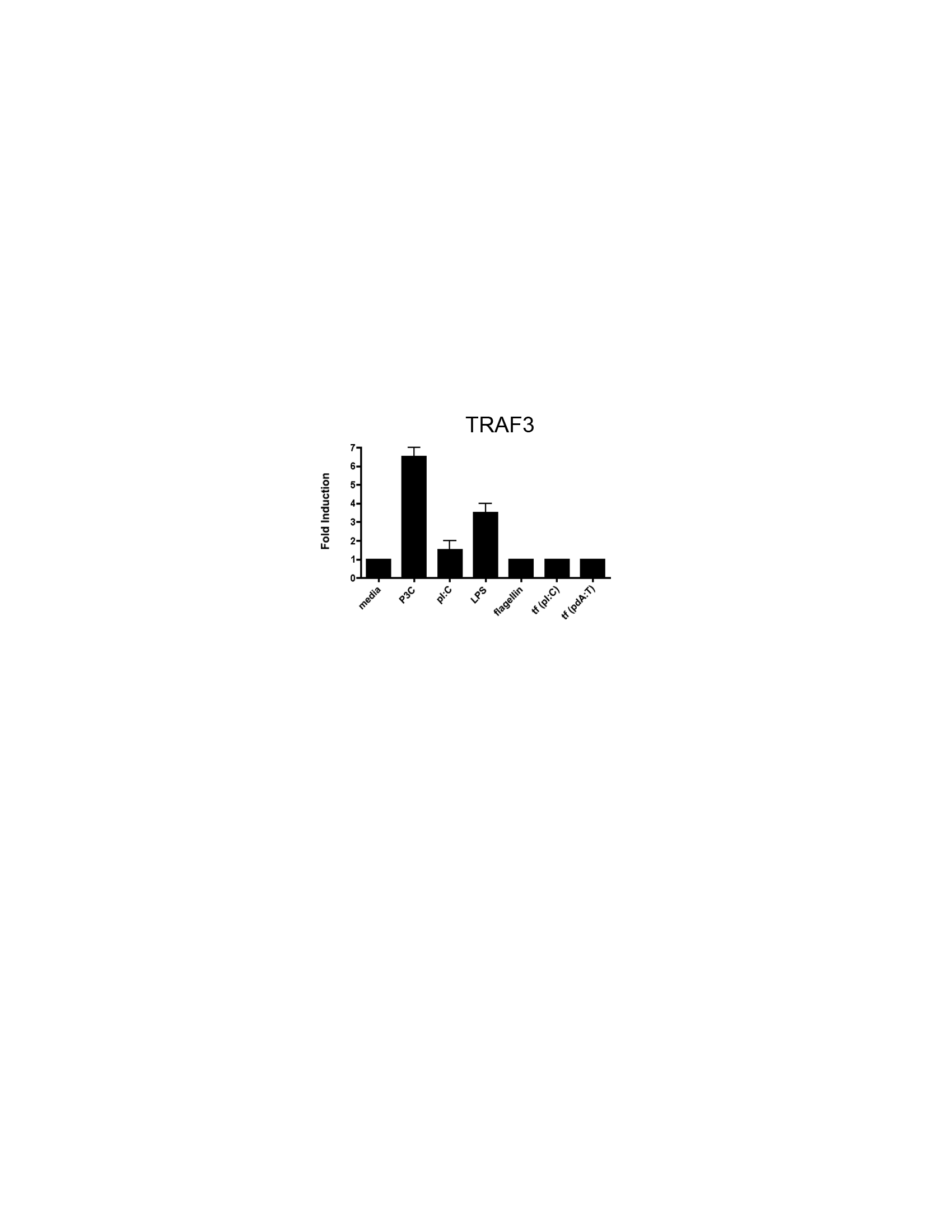

Supplement: Figure S5 — Differential TRAF3 induction by ligands of the innate immune system. Primary peritoneal macrophages were treated for 8 hours either with media alone or with Pam3Cys (250 ng/ml), soluble poly I:C (50 mg/ml), E. coli LPS (20 ng/ml), recombinant Salmonella flagellin (100 ng/ml), transfected poly I:C (500 ng/ml), or transfected poly dA:dT (500 ng/ml). Total RNA was harvested and used to analyze TRAF3 expression by qRT-PCR. (TIF) [file ppat.1003479.s005.tif]
